# Supplementary material for: Quantitative analysis of printed nanostructured networks using high-resolution 3D FIB-SEM nanotomography
Source: Nat Commun. 2024 Jan 4;15:278. doi: 10.1038/s41467-023-44450-1 (PMC10767099; doi:10.1038/s41467-023-44450-1)
Supplement: Supplementary file 1 — Supplementary Information [file 41467_2023_44450_MOESM1_ESM.pdf]

Supplementary information for

Quantitative analysis of printed nanostructured networks using high-resolution 3D FIB-SEM nanotomography

Cian Gabbett, Luke Doolan, Kevin Synnatschke, Laura Gambini, Emmet Coleman, Adam G. Kelly, Shixin Liu, Eoin Caffrey, Jose Munuera, Catriona Murphy, Stefano Sanvito, Lewys Jones, Jonathan N. Coleman*

*Corresponding author: colemaj@tcd.ie

Contents

Supplementary note 1. Basic nanosheet and network characterisation 2

Supplementary note 2. FIB-SEM Nanotomography 11

Supplementary note 3. Image segmentation 13

Supplementary note 4. Image stack alignment and 3D reconstruction 15

Supplementary note 5. FIB-SEM-NT spatial resolution and reduced volume analysis 17

Supplementary note 6. Network porosity & pore connectivity 20

Supplementary note 7. Network tortuosity factor measurements 24

Supplementary note 8. Pore size and shape 26

Supplementary note 9. Nanosheet aggregation within printed networks 31

Supplementary note 10. Nanosheet orientation 37

Supplementary note 11. Electrical characterisation of printed graphene networks 41

Supplementary note 12. Analysis of network surfaces and interfaces 44

Supplementary note 13. Depth Aware video frame INterpolation (DAIN) 48

Supplementary references 49

Supplementary note 1. Basic nanosheet and network characterisation

LPE graphene nanosheets and networks

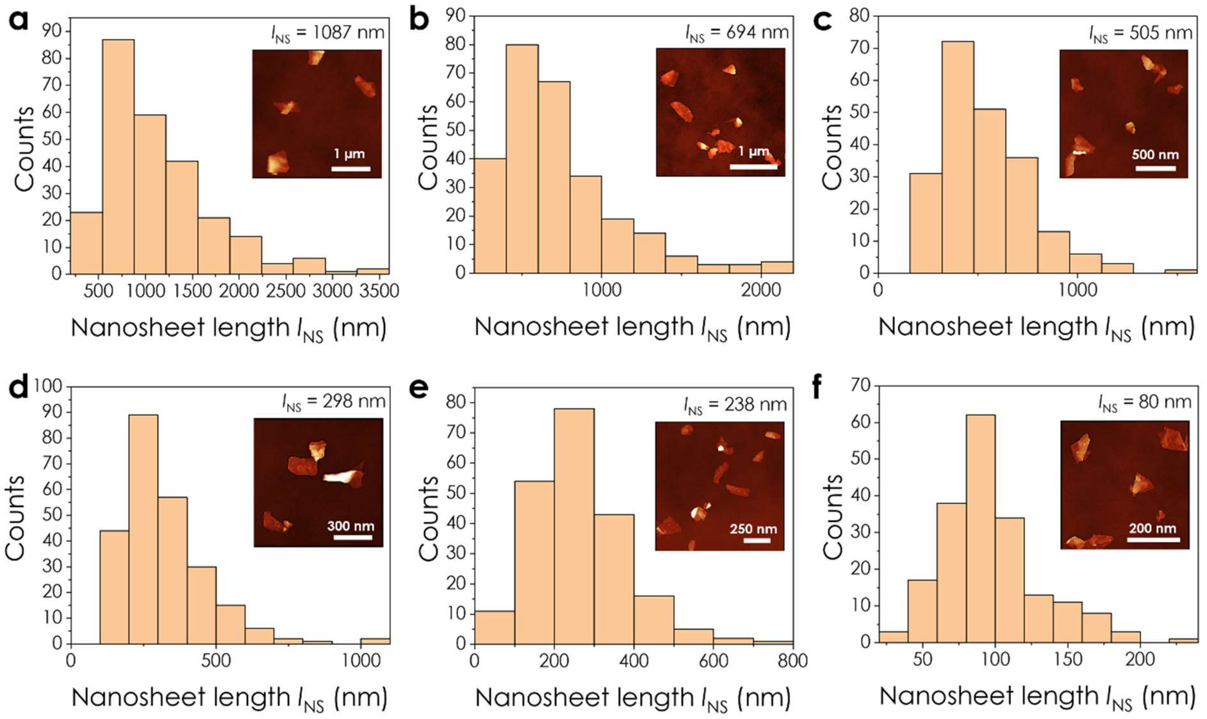

Supplementary figure 1: AFM Histograms for graphene nanosheet length. Distributions of nanosheet length, l_{NS}, measured using AFM for the size-selected LPE graphene inks. The distributions **(a-f)** are sorted by decreasing nanosheet length. Inset: Representative AFM images for each network.

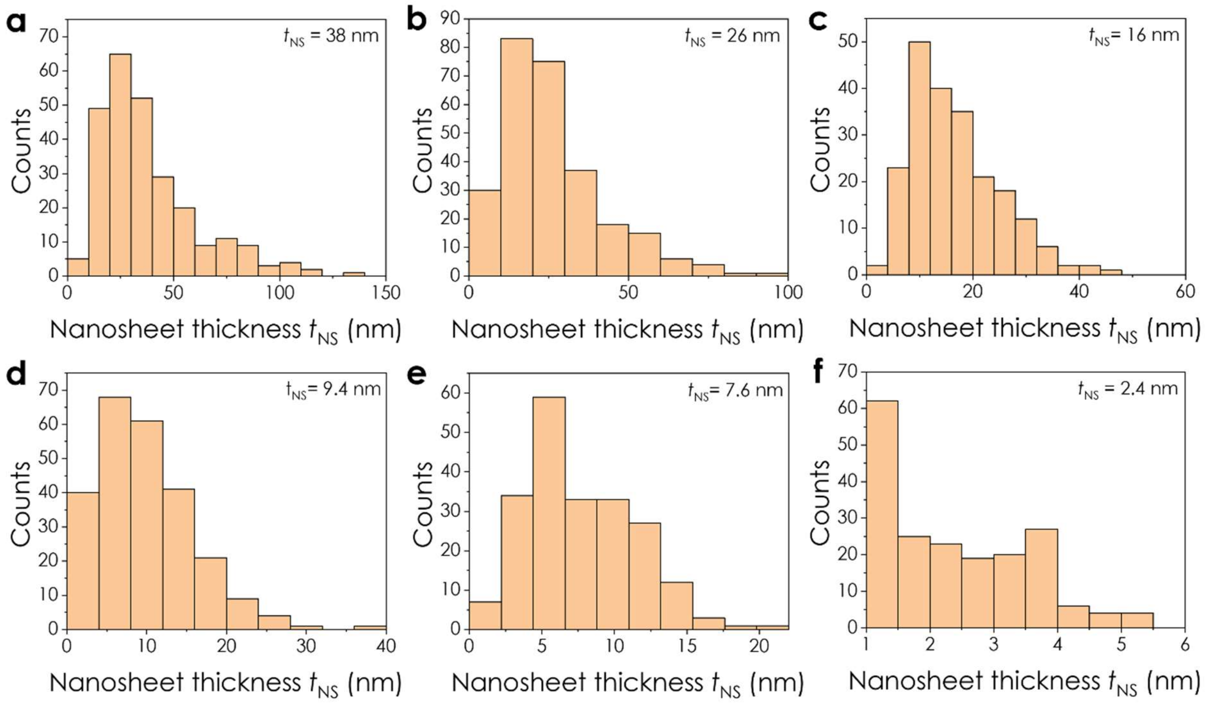

Supplementary figure 2: AFM histograms for graphene nanosheet thickness. Distributions of nanosheet thickness, t_{NS}, measured using AFM for the size-selected LPE graphene inks. The distributions **(a-f)** are sorted by decreasing nanosheet thickness.

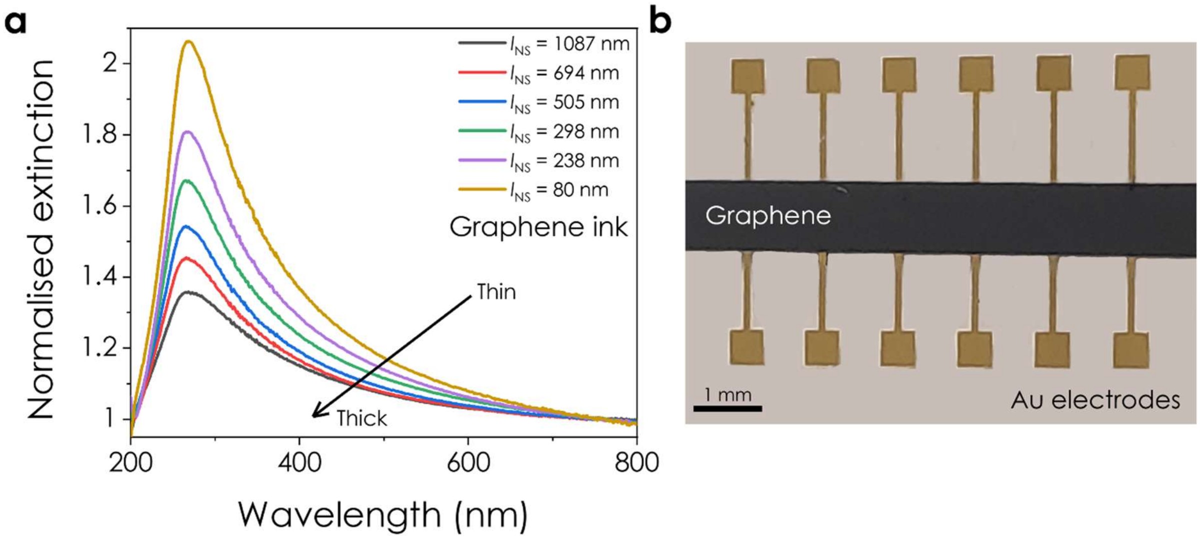

Supplementary figure 3: UV-vis of LPE graphene inks and deposition into a network trace. **(a)** Optical extinction spectra for the size-selected graphene inks normalised to the dimension independent plateau at 750 nm. **(b)** Spray-cast network of graphene nanosheets patterned onto a glass substrate using a shadow-mask. Evaporated gold bottom electrodes were used for electrical measurements.

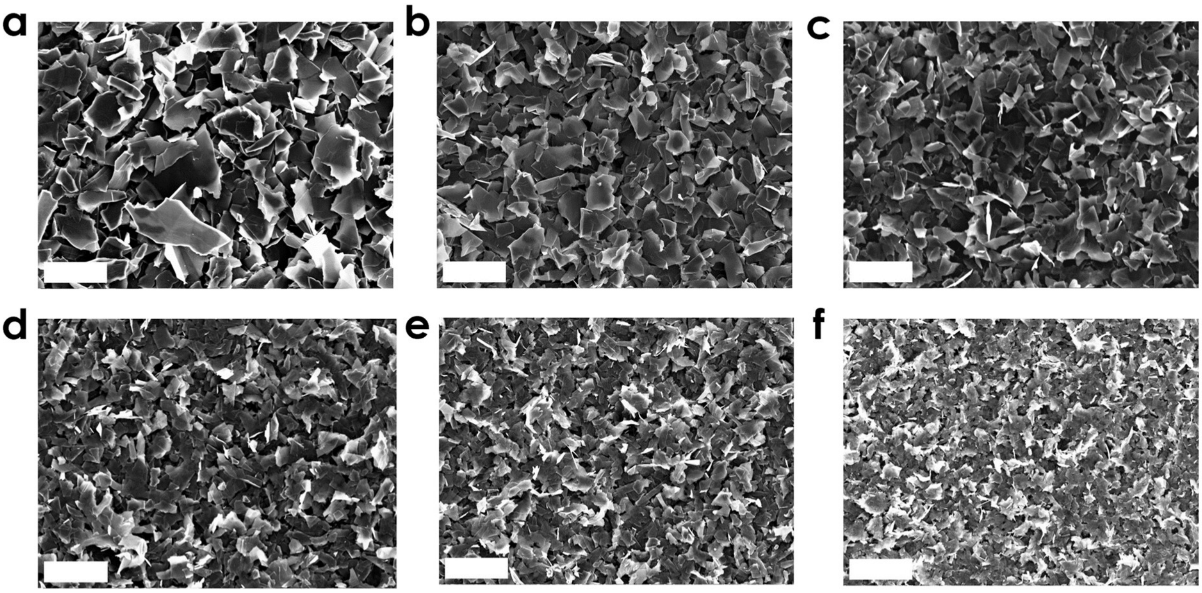

Supplementary figure 4: Surface SEM of printed LPE graphene networks. Surface SEM images of each size-selected sprayed graphene nanosheet network (Inlens detector) to qualitatively show changes in morphology with varying nanosheet size. The images in **(a-f)** are sorted by decreasing nanosheet length, l_{NS}. The scalebar on each image is 2 μm.

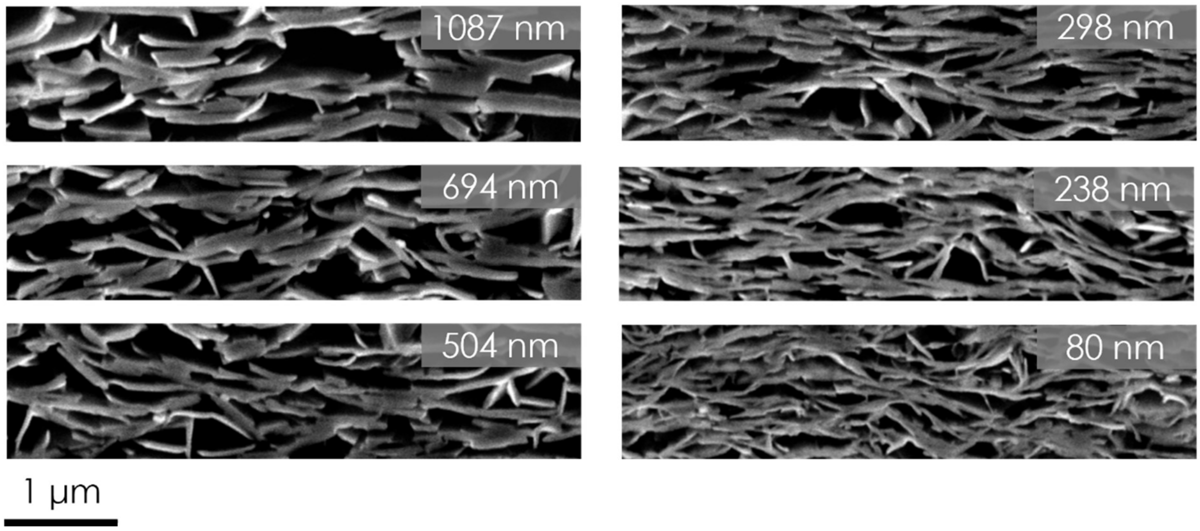

Supplementary figure 5: Cross-sectional SEM of printed LPE graphene networks. Cross-sectional SEM images of each size-selected sprayed graphene nanosheet network (SE2 detector) to qualitatively show changes in morphology with nanosheet length.

LPE WS_2 nanosheets and networks

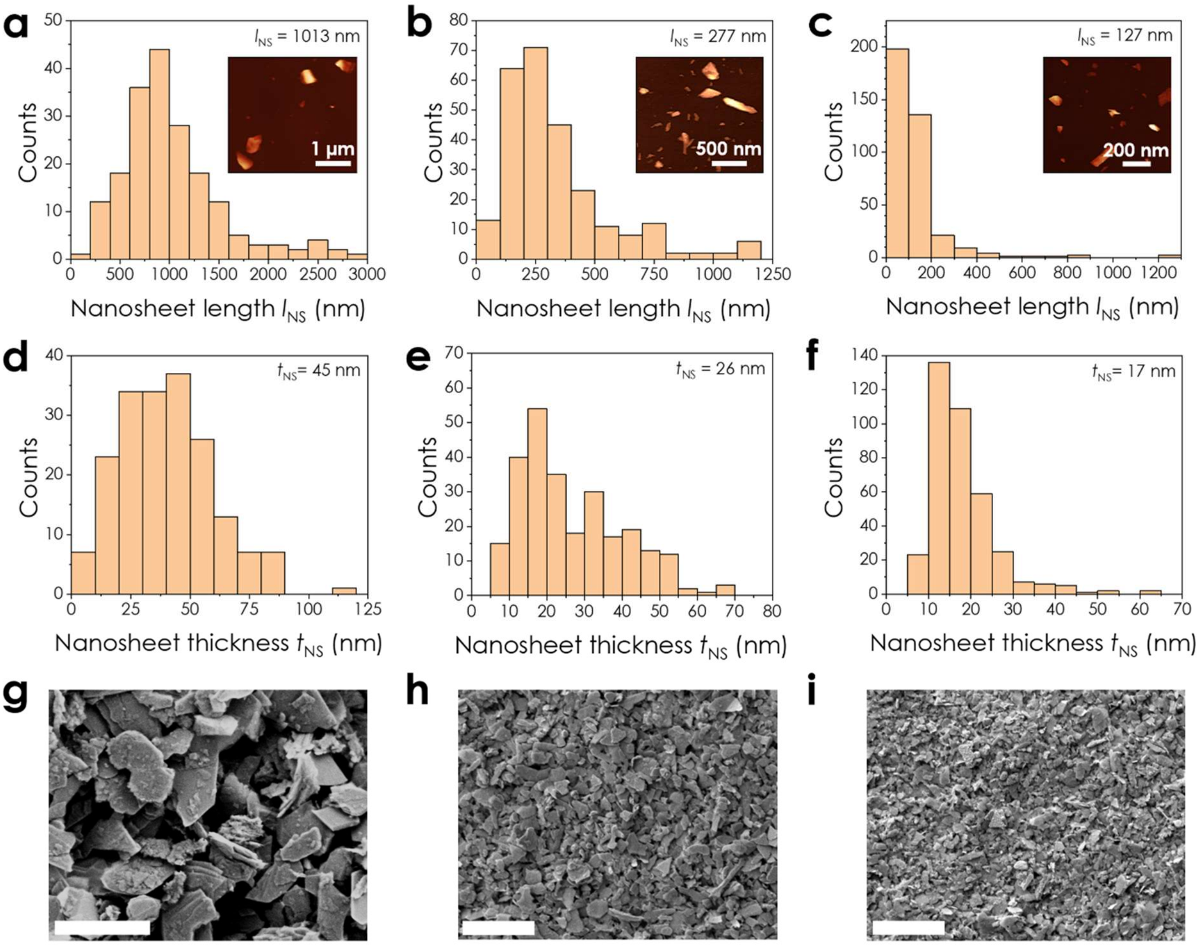

Supplementary figure 6: WS_2 nanosheet and network characterisation. Distributions of nanosheet **(a-c)** length, l_{NS}, and **(d-f)** thickness, t_{NS}, measured using AFM for the size-selected LPE WS_2 inks. The distributions **(a-c)** and **(d-f)** are sorted by decreasing l_{NS} and t_{NS}, respectively. Inset: Representative AFM images for each network. **(g-i)** Surface SEM images of each size-selected sprayed WS_2 network (Inlens detector) to qualitatively show changes in morphology with varying nanosheet size. The images in **(g-i)** are sorted by decreasing nanosheet length, l_{NS}. The scalebar on each image is 1 µm.

Silver nanosheets (AgNSs) and AgNS networks

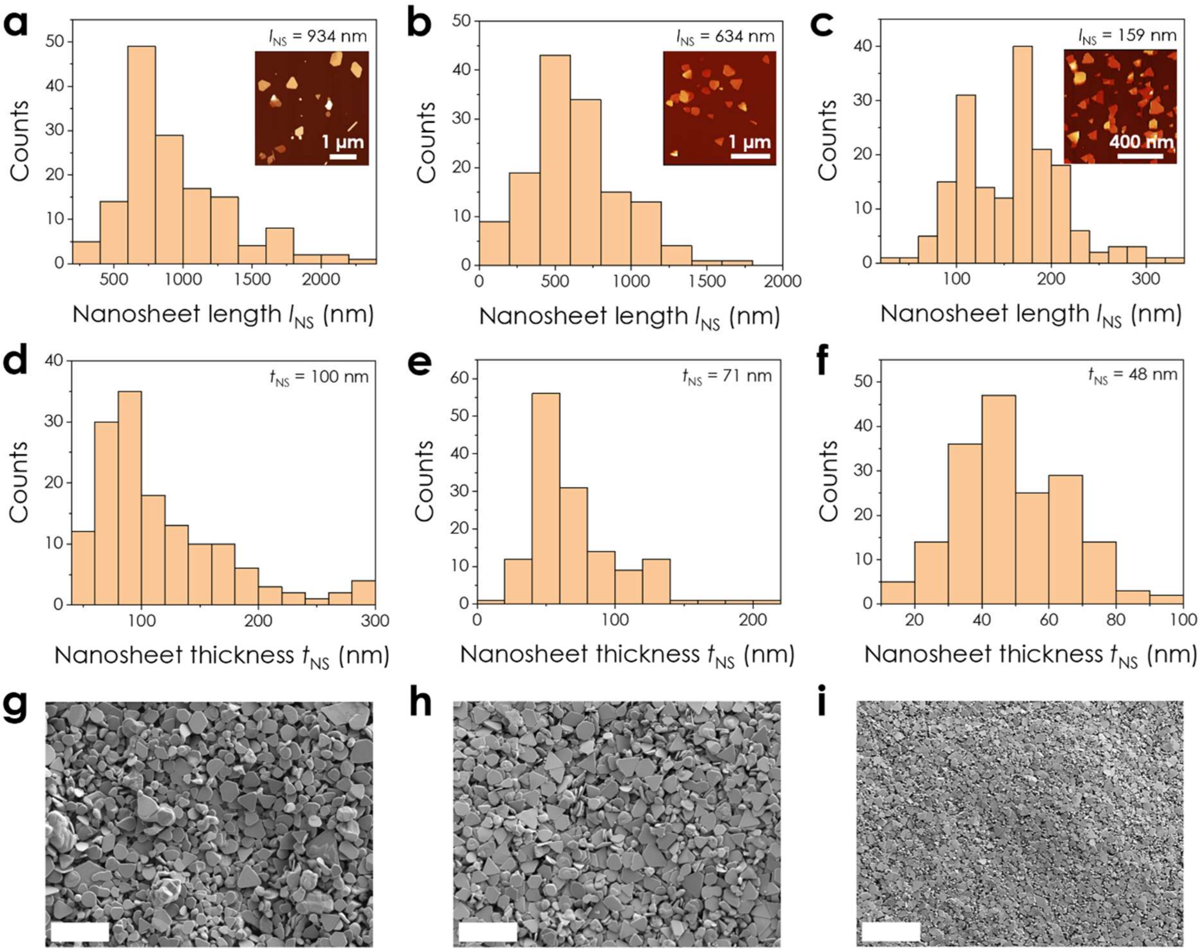

Supplementary figure 7: AgNS nanosheet and network characterisation. Distributions of nanosheet **(a-c)** length, l_{NS}, and **(d-f)** thickness, t_{NS}, measured using AFM for the size-selected AgNS inks. The distributions **(a-c)** and **(d-f)** are sorted by decreasing l_{NS} and t_{NS}, respectively. Inset: Representative AFM images for each network. **(g-i)** Surface SEM images of each size-selected sprayed AgNS network (Inlens detector) to qualitatively show changes in morphology with varying nanosheet size. The images in **(g-i)** are sorted by decreasing nanosheet length, l_{NS}. The scalebar on each image is 2 µm.

Silver nanowires (AgNWs) and AgNW networks

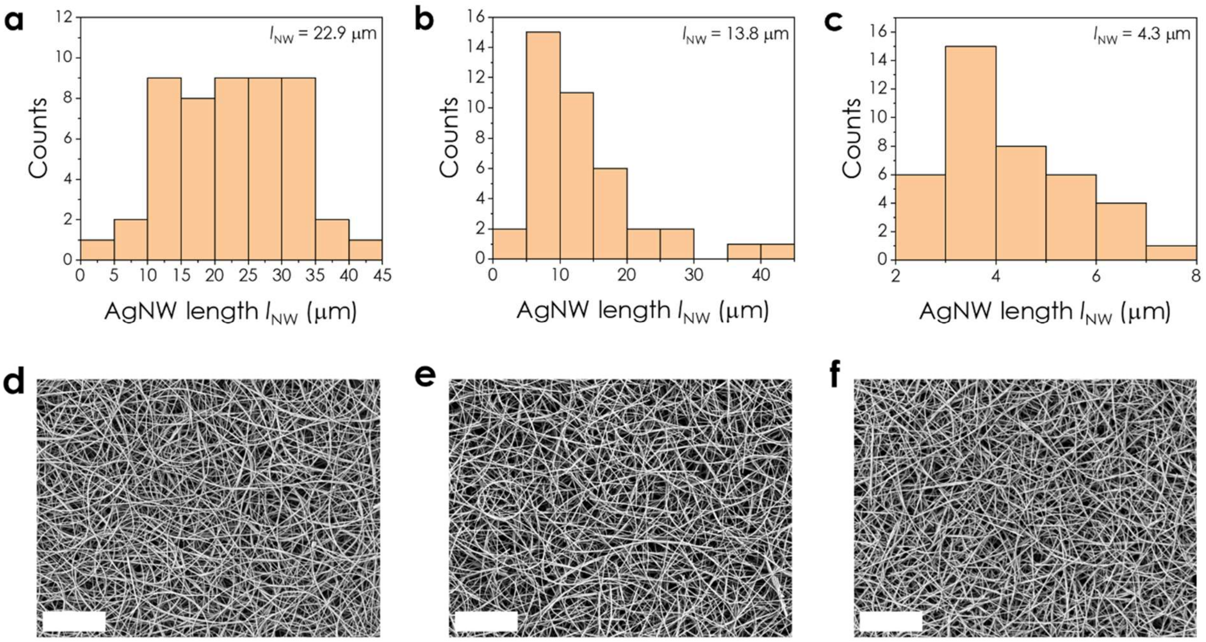

Supplementary figure 8: Characterisation of AgNWs and AgNW networks. **(a-c)** Distributions of nanowire length, l_{NW}, measured using SEM for the size-selected AgNW inks. The distributions **(a-c)** are sorted by decreasing nanowire length. **(d-f)** Surface SEM images of each size-selected sprayed AgNW network (Inlens detector) to qualitatively show changes in morphology with varying AgNW length. The images in **(d-f)** are sorted by decreasing nanowire length, l_{NW}. The scalebar on each image is 4 μm.

<u>LPE vs. EE graphene nanosheet and network comparison</u>

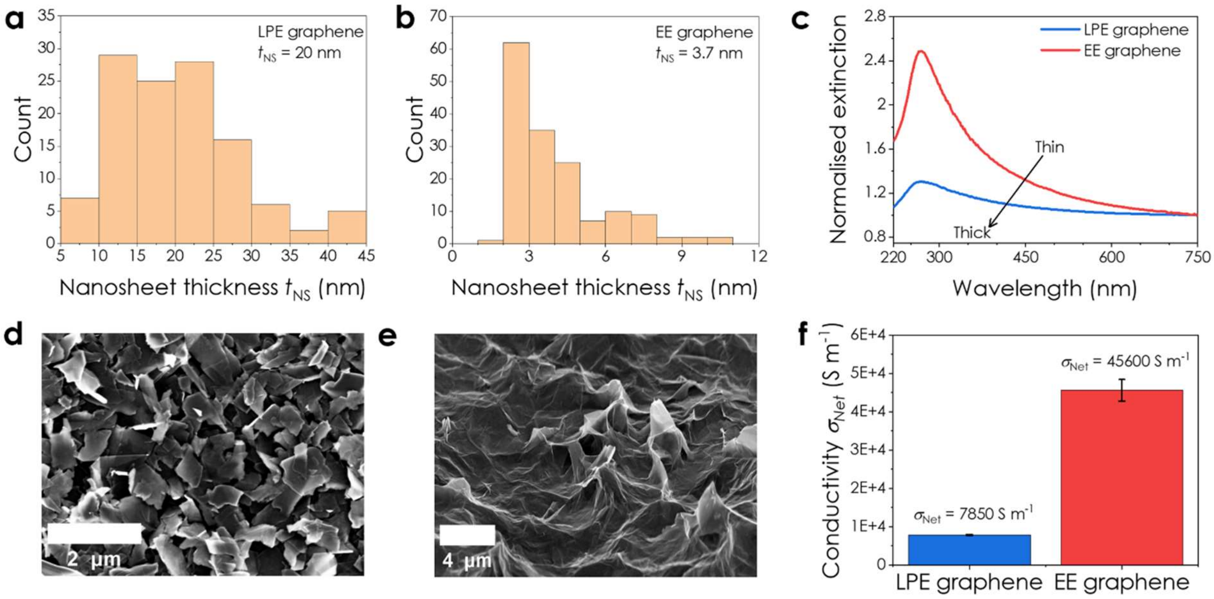

Supplementary figure 9: **Characterisation of EE and LPE graphene nanosheets and their networks.** Distributions of nanosheet thickness, t_{NS}, measured using AFM for the **(a)** LPE and **(b)** EE graphene inks. **(c)** Optical extinction spectra for the LPE and EE graphene inks normalised to the dimension independent plateau at 750 nm. **(d-e)** Surface SEM images of the sprayed **(d)** LPE and **(e)** EE graphene networks (Inlens detector). **(f)** Plot of the measured in-plane electrical conductivity, σ_{Net}, of the sprayed LPE and EE graphene networks. The data are presented as means ± standard error (SE) in the mean (n = 3).

Supplementary note 2. FIB-SEM Nanotomography

The FIB-SEM nanotomography (FIB-SEM-NT) in this work was performed using a dual beam Carl ZEISS Auriga FIB-SEM at an accelerating voltage of 30 kV. Sequential network cross-sections were automatically milled and imaged using the ZEISS ATLAS 5 software. The FIB-SEM-NT process is shown for a printed LPE graphene network in Suppl. Fig. 10. A protective platinum pad (20 × 15 μm, ≈ 1.5 μm thick) is first deposited over the region of interest to prevent sample damage (1 nA beam current) (Suppl. Fig. 10a, yellow box). A set of auto-tune and 3D tracking marks are then etched into the platinum pad using a 50 pA beam (yellow lines in Suppl. Fig. 10a). These etched traces are then filled in using tungsten (50 pA beam) (Suppl. Fig. 10b), which will offer contrast when imaged in the SEM. An additional layer of platinum is then deposited on the surface of the pad (1 nA beam) (Suppl. Fig. 10c). This layer protects the tungsten lines within the pad and increases the contrast between the tungsten and platinum. A large viewing trench and 2 side trenches on either side of the platinum pad are then sputtered using a 10 nA beam (red regions in Suppl. Fig. 10a). These are removed to allow the network cross-sections to be imaged by the SEM and to prevent material redeposition. A fine trench (blue rectangle in Suppl. Fig. 10a) is then sputtered using a 2 nA beam to reveal a network cross-section, as well as the tungsten tracking marks within the platinum pad (Suppl. Fig. 11).

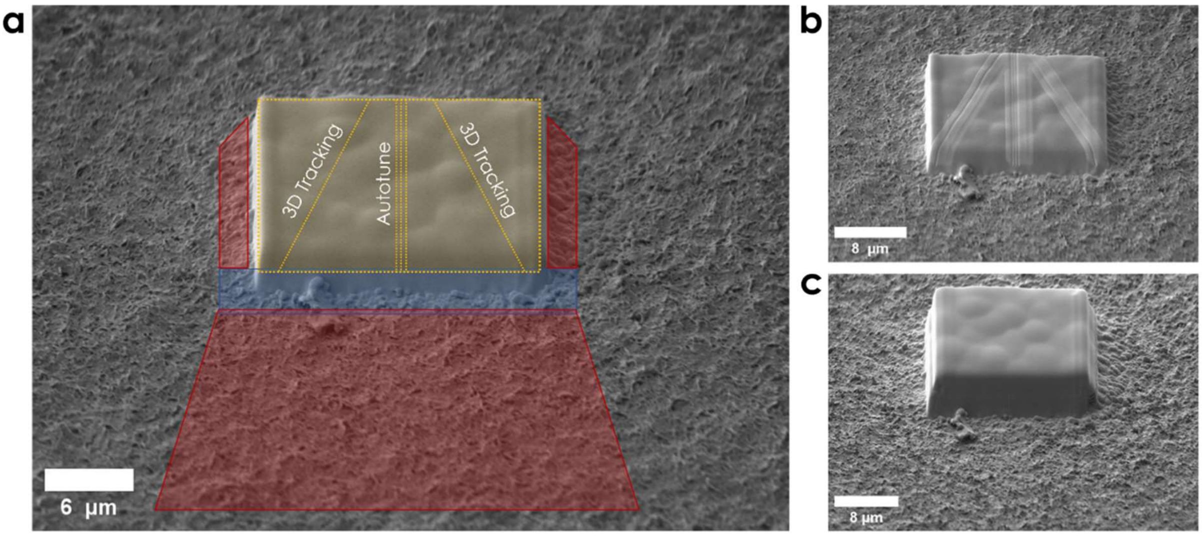

Supplementary figure 10: FIB-SEM-NT setup. **(a)** SEM image of a printed LPE graphene network with a platinum pad deposited on top. The region of interest is shaded yellow, while the viewing and fine trenches are shaded red and blue respectively. The 3D tracking and autotune marks are given by the dashed yellow lines. **(b)** SEM image showing a platinum pad with the 3D tracking and autotune marks etched into it and filled with tungsten. **(c)** SEM showing the pad after the surface had been covered with platinum to protect the 3D tracking and autotune marks.

The ATLAS 5 system stays focused and calibrated throughout the FIB-SEM-NT run by using the autotune reference marks. These are the three parallel lines shown in Suppl. Figs. 10 & 11. After every 10th network cross-section has been milled and imaged, the system finds the autotune marks and automatically adjusts both the SEM focus and stigmation until they are in sharp focus. This ensures the network cross-sections remain in focus throughout the run, as the system drifts and the working distance increases.

The angled 3D tracking lines (Suppl. Figs. 10 & 11) are used to measure the thickness of each slice removed from the network. The tungsten-filled lines are milled at a well-defined angle with respect to the system coordinates. As slices are removed from the network, the horizontal approach of the 3D tracking lines is measured to determine the slice thickness. The tracking marks also ensure that the slice thickness remains approximately constant throughout the process by correcting drifts in the FIB beam.

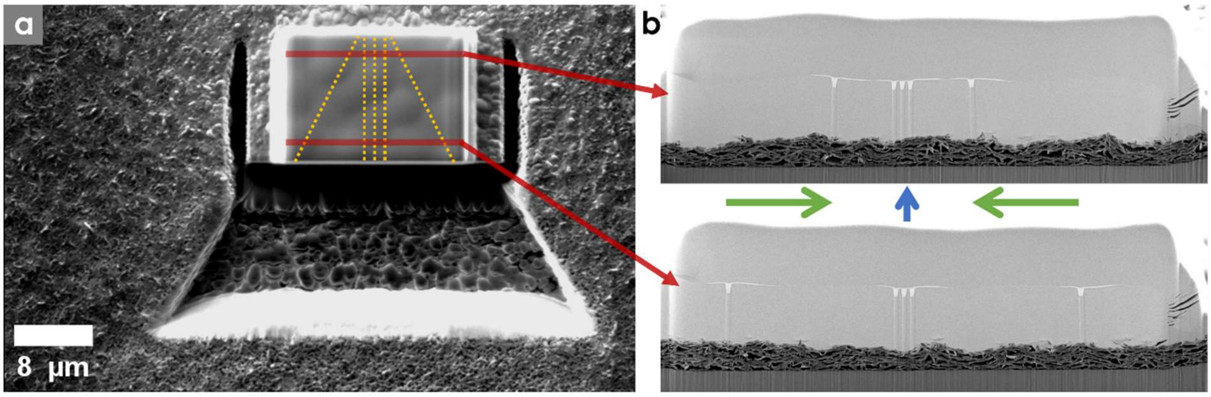

Supplementary figure 11: FIB-SEM-NT 3D tracking. **(a)** FIB image of a printed LPE graphene network prepared for a FIB-SEM-NT run (50 pA beam current). The 3D tracking and autotune marks are denoted by dashed lines. **(b)** SEM images of network cross-sections showing the horizontal approach of the 3D tracking lines as the milling depth through the sample is increased.

During the FIB-SEM NT run, the cross-section face was milled with a 600 pA beam and the average thickness of a slice removed was ≈ 15 nm. SEM images of the network cross-sections were captured using both Inlens and SE2 detectors (dwell time of 0.7 µs and a line average of 6), with a pixel size of 5 nm. The SEM contrast and brightness was tailored for each detector to maximise the difference in contrast between the material and pores.

Supplementary note 3. Image segmentation

Network cross-sections were segmented into their discrete phases using the trainable WEKA segmentation plugin in FIJI[1,2]. All cross-sectional images were captured using the SE2 detector at an accelerating voltage of 2 kV. This served to reduce the imaging depth of field into pores[3], alleviate charging effects[4] and increased topographic contrast between the nanosheets and pores when compared to the Inlens detector[5] (Suppl. Fig. 12a-b). Prior to applying the WEKA classifier, the pixel intensity in each network image was normalised. The image brightness and contrast were then adjusted globally across the entire stack using FIJI[2] to ensure maximum contrast between the different phases. This led to improved image classification and reduced the depth of field, which ensured that only nanosheets/pores at the cross-section face were considered for segmentation (Suppl. Fig. 12c).

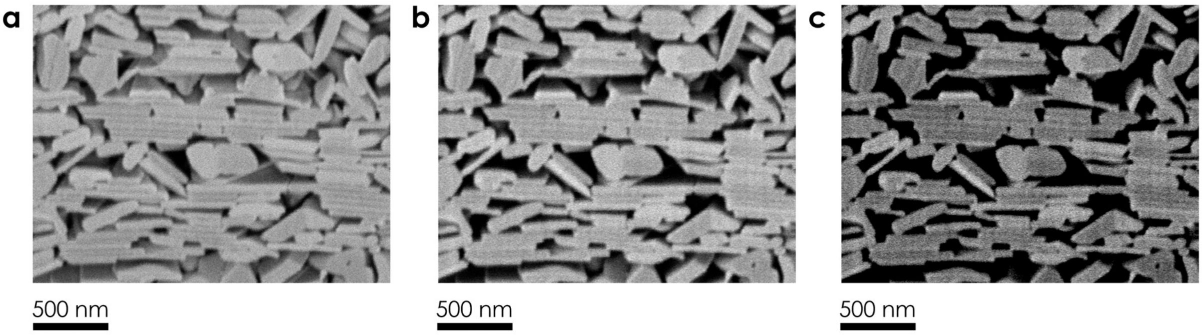

Supplementary figure 12: Detector choice and image pre-processing. High magnification cross-sectional SEM images of the same region of a printed silver nanosheet network. Images **(a)** and **(b)** were captured using the Inlens and SE2 detectors respectively. Image **(c)** was captured using the SE2 detector and the brightness/contrast was adjusted to reduce the shine-through effect and improve classification of the nanosheets and pores.

The WEKA classifier was initially trained on a representative subset of cross-sections from a given image stack. Regions of each image were first manually assigned as either "pore" or "nanosheet" to provide training data for the classifier (Fig. 1d, main text). The algorithm then segments the image with user-determined classification features that include edge detectors, texture filters and noise reduction filters. To ensure sharp boundaries between the different phases several edge-detecting features were employed including Sobel filters, Hessian matrix eigenvalues and the differences of Gaussians. Textural features were used to calculate the mean, variance, and maxima of pixels within a given radius of a target pixel and set the pixel to that value, as were bilateral filters. Gaussian blur filters were applied to reduce imaging artefacts and local intensity variations in an image. The classification algorithm used was a multi-threaded random forest classifier with 200 uncorrelated decision trees and two random features per node[6]. Such classifiers have shown comparable performance to manual segmentation by an expert for porous polymer networks[7].The performance of the classification process was assessed through the out-of-bag (OOB) error, which is a measure of how well decision trees in the model perform in classifying subsets of data that they were not directly trained on. For each network in this work the classification algorithm was trained until the OOB error was ≈ 2%.

The result of the segmentation process is a stack of probability maps, which are network cross-sections where the greyscale pixel intensity has been replaced by the probability that each pixel belongs to a given class (i.e. nanosheet or pore). These were then binarised using the Isodata algorithm in FIJI[8] to produce stacks of nanosheet-only and pore-only images for each network (Fig. 1d, main text). WEKA trainable segmentation can be applied to >2 phases in an identical manner, as shown for the printed heterostacks in Fig. 6b-c in the main text.

Metrics such as the Jaccard index can be used to estimate segmentation error by comparing segmented and hand-annotated ground-truth images. However, manually generating a representative subset of annotated ground-truth images for stacks of hundreds of images was not possible. Other strategies include selectively thresholding the probability maps at values corresponding to ± 1 standard deviation about a central value to construct percentile segmentation domains and estimate the bounds of uncertainty[9]. However, this did not produce reasonable results here, and the selection of intermediate percentile values was not attempted as it would have been arbitrary. In this work, the OOB classification error is used to estimate the segmentation uncertainty in the binarised volumes.

Supplementary note 4. Image stack alignment and 3D reconstruction

The product of a FIB-SEM NT run is a stack of hundreds of network cross-sections where the thickness of each slice was measured during the process. However, due to drifts in both the SEM and FIB during a run, images in a stack may be vertically/horizontally shifted with respect to each other (Suppl. Fig. 13a). Such misalignment would be detrimental to any attempt to convert the stack into a representative 3D volume. We utilised the Slice Registration suite in Dragonfly (Version 2022.1.0.1231, Object Research Systems) to align the image stacks. Dragonfly has been used extensively to reconstruct tomographic data across the fields of materials science[10], life sciences[11], geosciences[12], and manufacturing. The sum of squared differences (SSD) matching process was used to align adjacent slices and a linear drift compensator was employed to correct systematic drifts in the process. Within Dragonfly there are several different algorithms that can be used to align an image stack (enhanced correlation coefficient, feature based, mutual information, SSD and template matching). We tested each algorithm on a silver nanosheet (AgNS) network volume (Suppl. Fig. 13b-f), finding the resultant network properties (porosity, tortuosity, and specific surface area) to be in broad agreement (Suppl. Fig. 13g). However, the best performance in terms of computational cost was achieved using the SSD matching process, so this algorithm was employed.

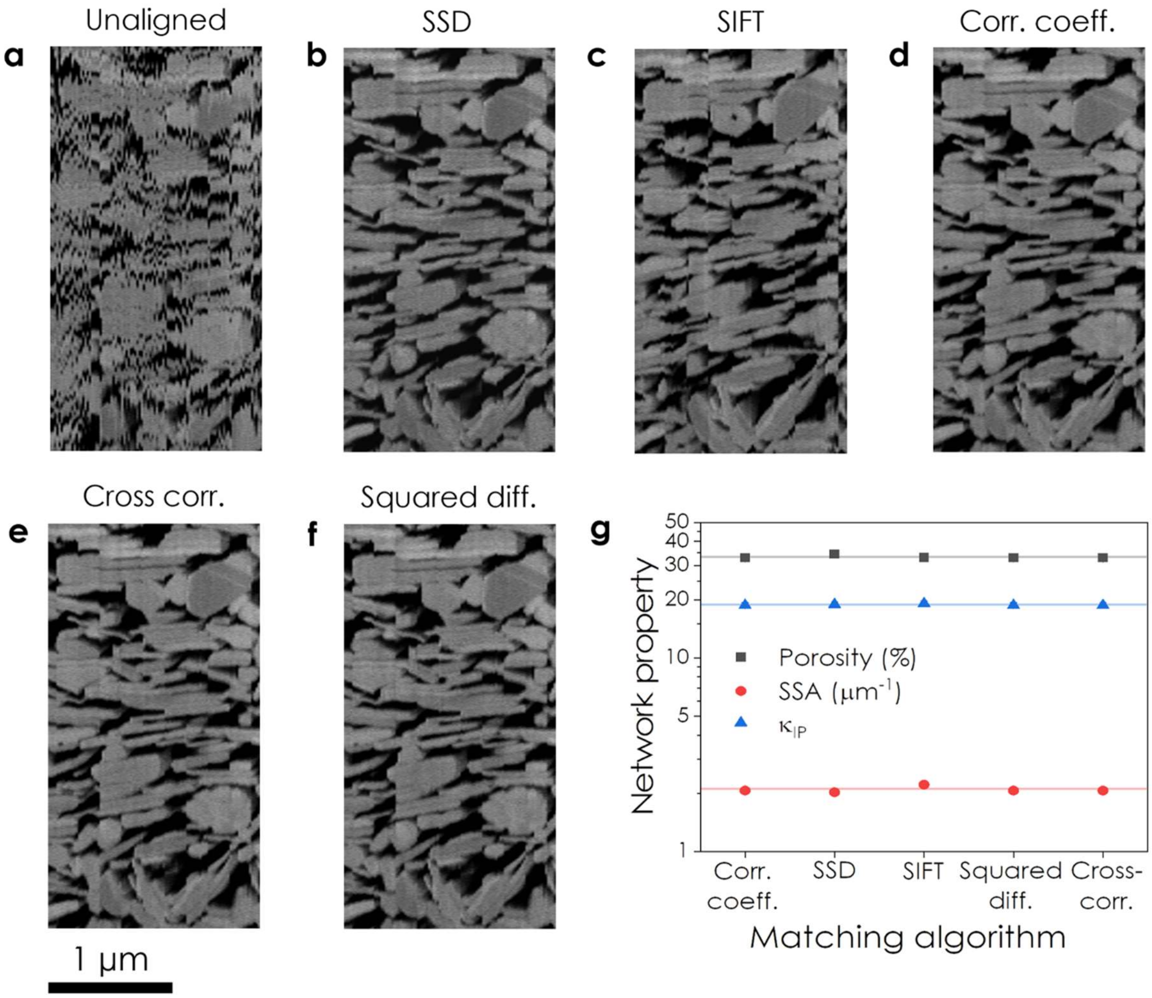

Supplementary figure 13: Image stack alignment for silver nanosheets (AgNSs) in Dragonfly. **(a)** The original, unaligned image stack, viewed in the *yz*-plane. Images were captured in the *xy*-plane. **(b-f)** The same image stack aligned using the **(b)** Sum of squared differences (SSD), **(c)** Scale-invariant feature transform (SIFT), **(d)** Correlation Coefficient, **(e)** Cross-correlation and **(f)** Squared difference matching algorithms. **(g)** Measured network porosity, tortuosity (in the *xy*-direction) and specific surface area (SSA) for each alignment method.

A representative SEM cross-section (SE2 detector) of a printed LPE graphene network in the slicing direction (*xy*-plane) is shown in Suppl. Fig. 14a (top row), with a pixel size of 5 nm. An entirely reconstructed image of the network in the perpendicular direction (*yz*-plane) shows severe misalignment of the slices, due to instabilities during the FIB-SEM-NT process (Suppl. Fig. 14a, middle row). This image stack was aligned in Dragonfly as described above to produce a reconstructed image of the network in the *yz*-plane (Suppl. Fig. 14a, bottom row). Despite the anisotropic pixel size (5 × 15 nm, due to the slice thickness) this image compares extremely well with the experimentally imaged network cross-section. Similarly, the network can be interrogated in the out-of-plane (*xz*-plane) direction, with the effect of correct slice alignment shown in Suppl. Fig. 14b. All reconstructed 3D volumes shown in the main text (Fig. 1e-f, Fig. 2a, Fig. 3b, Fig 5a-c and Fig. 6a-c) were generated using Dragonfly.

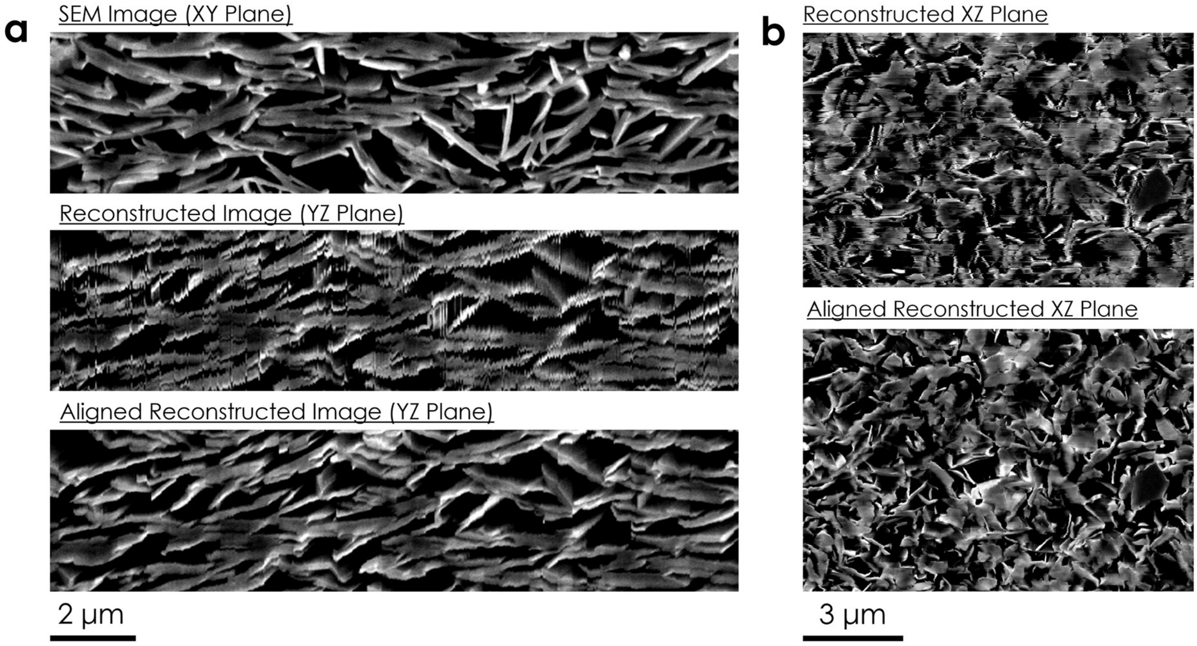

Supplementary figure 14: Image stack alignment for graphene nanosheets. **(a)** SEM cross-section of a printed LPE graphene network in the slicing (*xy*-plane) direction (top row). A reconstructed view of the network in the *yz*-plane is shown before (middle row) and after (bottom row) slice alignment has been performed. **(b)** Reconstructed view of the same network in the *xz*-plane before and after slice alignment.

Supplementary note 5. FIB-SEM-NT spatial resolution and reduced volume analysis

To highlight the resolution advantage of FIB-SEM-NT for accurate reconstruction of printed nanosheet networks we imaged a vacuum filtered silver nanosheet (AgNS) film using a lab-based X-ray micro CT microscope (Nikon XTH 225, voxel size = 4 × 4 × 4 µm). The AgNSs were vacuum filtered to ensure the film was thick enough to be imaged (thickness = 80 µm) and because the contrast between the silver nanosheet network and the filter membrane was sufficient to allow visualisation of the network. The voxel size of 4 µm is much larger than both the nanosheet length (l_{NS} = 634 nm) and thickness (t_{NS} = 71 nm), so no information on the internal structure of the network is obtained. A printed network of the same silver nanosheets that has been reconstructed using FIB-SEM NT is shown in Fig. 15b (and Fig. 5b, main text). Here, the enhanced resolution (voxel size = 5 × 5 × 15 nm) allows individual nanosheets to be resolved, as well their orientation and dimensions.

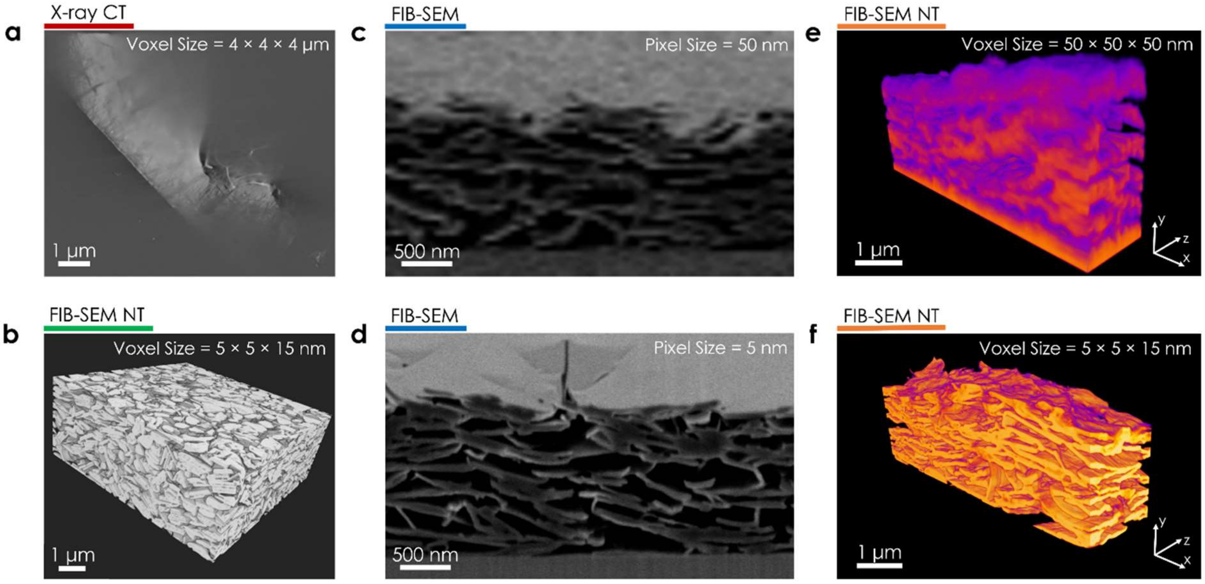

Supplementary figure 15: Network structure for different imaging techniques and pixel/voxel sizes. **(a)** Image of a vacuum filtered silver nanosheet (AgNS) network on a filter membrane captured using an X-ray micro CT scanner with a voxel size of 4 × 4 × 4 µm. **(b)** A printed network of the same AgNS reconstructed using FIB-SEM-NT with a voxel size of 5 × 5 × 15 nm. **(c-d)** FIB-SEM cross-sections of a printed graphene network captured using a pixel size of **(c)** 50 nm and **(d)** 5 nm. **(e-f)** Reconstructed volumes of the same graphene network using voxel sizes of **(e)** 50 × 50 × 50 nm and **(f)** 5 × 5 × 15 nm. The pixel and voxel sizes used in **(c)** and **(e)** are representative of the X-ray nano CT technique.

A significant drawback to X-ray nano CT is that the technique is highly specialised and not widely accessible. Thus, we captured FIB-SEM cross-sections of the same printed graphene network at a pixel size of 50 nm, as a proxy for the typical resolution achievable using nano CT (Suppl. Fig. 15c), and at the pixel size used in this work of 5 nm (Suppl. Fig. 15d). The loss of information in Suppl. Fig. 15c is clear when compared to the image in Suppl. Fig. 15d, where the pixel size is larger than the nanosheet thickness in many cases. This causes the interphase boundaries (nanosheet/pore/substrate/platinum) to become blurred, which translates to a very poor reconstruction of the network structure in Suppl. Fig. 15e. Such volumes would clearly not offer accurate morphological information and highlights the need for the spatial resolution FIB-SEM NT can provide, as shown for the same network in Suppl. Fig. 15f.

Cryogenic electron tomography (3D-TEM) has a superior spatial resolution to FIB-SEM-NT at the cost of imaging a much smaller volume. Samples for 3D-TEM must be electron transparent, meaning the volumes to be analysed must be less than ≈500 nm thick[13]. This thickness requirement, as well as the tilting process for 3D-TEM, limits the imaged volumes to be on the order of ≈1 μm^3 in size[14]. Such small network volumes would not be expected to be representative of a complete nanostructured network or device. Many of the nanosheets in this work are > 500 nm long, while the AgNW length in the networks analysed range between 4.3 – 22.9 μm. Thus, morphological properties that we calculate in the main text including network porosity, pore shape/size, nanosheet/pore tortuosity and specific surface area cannot be determined accurately using 3D-TEM. For example, we show in the main text (Fig. 2b) that the measured porosity from a single network cross-section (≈ 0.53 μm^3) can vary by as much as ± 10% from the average value across the entire network volume. Properties such as pore connectivity and tortuosity are far more severely affected by analysis of small volumes. To show this, we performed reduced volume analysis on a printed graphene network (l_{NS} = 298 nm) in Suppl. Fig. 16. Here, the tortuosity factor of the pore volume was measured in the in-plane z-direction (the slicing direction) as a function of the considered network volume.

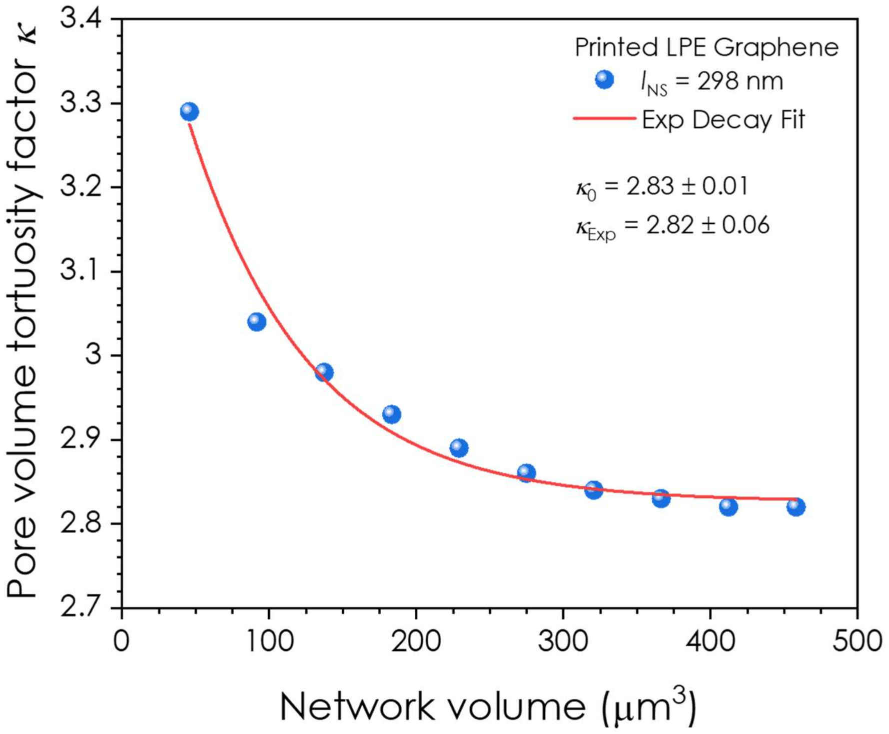

Supplementary figure 16: Reduced volume analysis for the pore tortuosity factor in a printed graphene network. Plot of the measured the tortuosity factor, κ, of the pore volume in the in-plane z-direction (the slicing direction) for a network of printed LPE graphene nanosheets (l_{NS} = 298 nm) as a function of the sampled network volume. The red line is a fit to a single exponential decay. κ_0 is the fitted plateau value for the tortuosity factor and κ_{Exp} is the measured value for the full volume imaged using FIB-SEM-NT.

Suppl. Fig. 16 shows that for smaller network volumes the measured tortuosity factor is overestimated, likely due to the reconstructed volume being too small for isolated pores to coalesce. However, we see κ decrease with increasing sample volume as the discrete pores become connected and that κ stabilises to a plateau for values greater than ≈ 200 μm^3 (*cf.* sampled volumes of 1 μm^3 for 3D TEM). This highlights that while 3D TEM may offer higher resolution than FIB-SEM-NT, the sampled volumes are likely too small to fully describe the morphology of printed nanosheet/nanowire networks and their devices. The data in Suppl. Fig. 16 also demonstrates that while X-ray nano CT can be used to probe larger sample volumes, the achievable volumes of 10^2 – 10^3 μm^3 using FIB-SEM-NT are large enough to be representative of the overall network.

Supplementary note 6. Network porosity & pore connectivity

The pore connectivity within the networks was quantified using the Find Connected Regions plugin in FIJI[2]. This plugin calculates the number of voxels within each discrete pore in a binarized 3D volume, and searches for connections between adjacent pores. The calculations were performed allowing connections between vertices and with the minimum number of voxels in a region set to 2. When pore connectivity was measured across entire network volumes, > 99% of the total pore volume was found to be part of contiguous macropore spanning the volume for most networks considered in this work. However, by considering reduced network volumes (Suppl. Fig. 17a) it was possible to assess the pore connectivity as a function of the network dimensions. The pore connectivity was quantified by the fraction of the global pore volume contained within the largest pore in that volume.

The increase in pore connectivity as the network volume is increased from 1 slice (≈15 nm) to ≈ 20 slices (≈300 nm) is shown for 7 different portions of a printed LPE graphene network (l_{NS} = 238 nm) in Suppl. Fig. 17b. The average depth into the 3D volume required for 95% of the porosity to be contained within a single connected pore, which we define as the connectivity length scale in the main text, was 251 ± 9 nm (or a volume of 8.94 ± 0.3 μm^3).

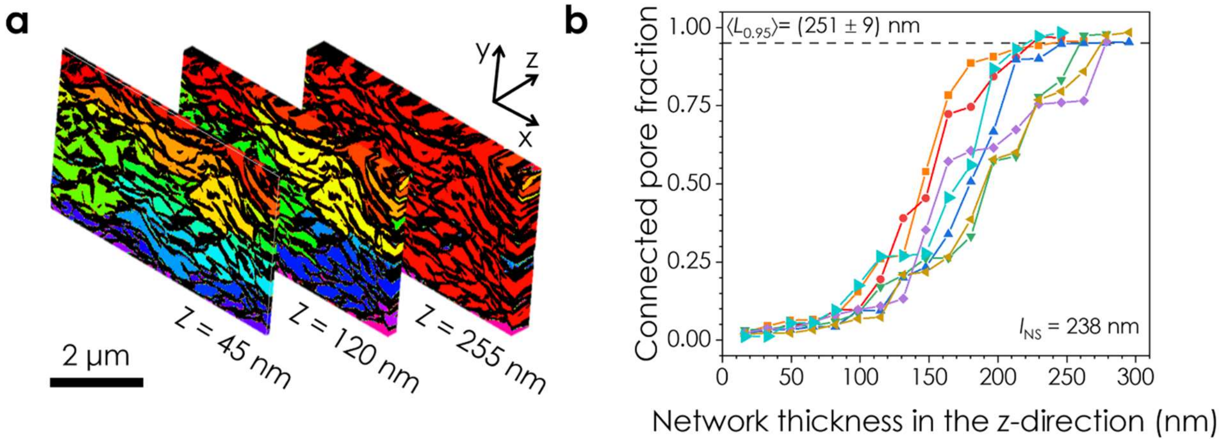

Supplementary figure 17: Reduced volume connectivity analysis. **(a)** Pore connectivity in reduced network volumes of thickness z = 45, 120 and 255 nm for a printed LPE graphene network (l_{NS} = 238 nm). As the thickness of the interrogated volume increases, the colour-coded discrete pores coalesce into a connected (red) macropore. Nanosheets are coloured black. **(b)** Increase in the connected pore fraction as the number of slices (network thickness) is increased for the l_{NS} = 238 nm printed graphene network. Each line denotes a different region of the network measured. The dashed black line marks a connected pore fraction of 0.95.

Local porosity variations in the networks were characterised by reslicing the 3D images in the *x*,*z* (in-plane) and *y* (out-of-plane) directions and measuring the 2D porosity on a slice-by-slice basis in each direction. This is shown for the printed LPE graphene networks in Suppl. Fig. 18. The porosity vs. slice data in the *x*,*z* directions exhibit local variations in porosity (Suppl. Fig. 18a-b), with an average standard deviation of ≈4% from the global mean. This suggests that individual FIB-SEM cross-sections may not be sufficient to accurately quantify the network porosity. Interestingly, each network exhibits larger porosities at the base of the network, with the porosity of the sample decreasing as slice number is increased in the *z*-direction away from the substrate. (Suppl. Fig. 18c).

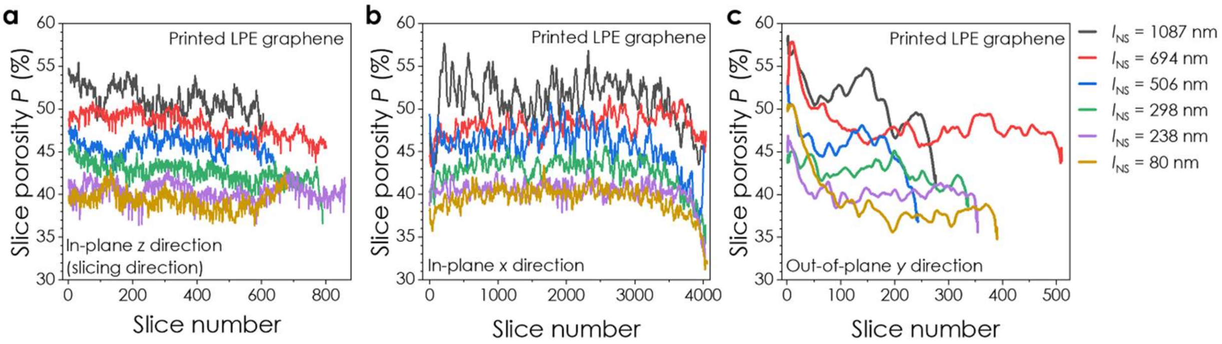

Supplementary figure 18: Porosity vs. slice data in the *x*, *z*, and *y*-directions for the sprayed graphene networks. **(a)** Porosity, *P*, as a function of slice number in the direction the network was milled (*z*-direction, in-plane). **(b)** Porosity as a function of slice number perpendicular to the slicing direction (*x*-direction, in-plane). **(c)** Porosity as a function of slice number in the *y*-direction (out-of-plane).

The porosity vs. slice data show similar trends in each direction for the 2D WS_2 (Suppl. Fig. 19a-c) and AgNS (Suppl. Fig. 19d-f) networks. Here, the slice-by-slice porosity in the in-plane (*x*,*z*) and out-of-plane *y*-directions exhibit considerable scatter about the global average. The 1D AgNW networks show comparable scatter about the overall average in the in-plane (*x*,*z*) directions (Suppl. Fig. 19g-h). However, there is a strong trend in the slice-by-slice porosity in out-of-plane (*y*) direction (Suppl. Fig. 19i). The porosity appears to decrease approximately linearly with AgNW network thickness (increasing slice number, or distance from the substrate), which suggests the porosity of these AgNW networks are thickness dependent. Interestingly, the data in Suppl. Fig. 19i suggests that as the AgNW length is reduced, the rate at which the network porosity decreases is increased. This suggests that the decrease in porosity in the *z*-direction is related to nanowires filling in open spaces in underlying layers within the network.

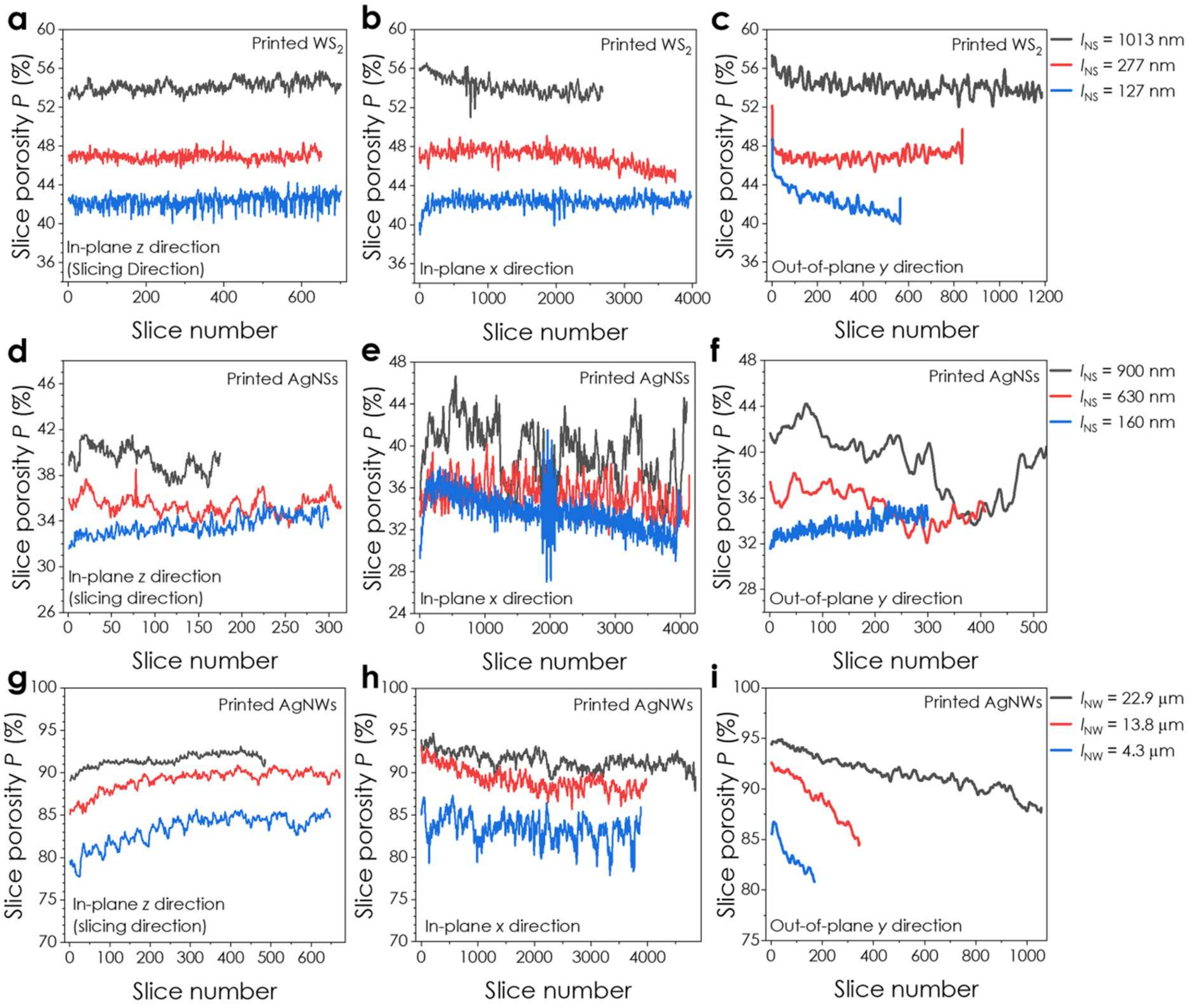

Supplementary figure 19: Porosity vs. slice data in the *x*, *z* and *y*-directions for the sprayed WS_2, AgNS and AgNW networks. Porosity, P, as a function of slice number in the x,z (in-plane) and y (out-of-plane) directions for the sprayed **(a-c)** WS_2, **(d-f)** AgNS and **(g-i)** AgNW networks.

The porosity of a network of randomly packed rigid 1D fibres has been modelled by Parkhouse et al.[15] as

$$P = 1 - \frac{2\ln(k)}{k} \quad (1)$$

Where P is the porosity of the network and k is the fibre aspect ratio (length/diameter). This has been shown to apply to networks of copper nanorods (diameter ≈ 220 nm)[16]. However, while this equation describes the porosity scaling in size-selected AgNW networks well (diameter ≈ 55 nm), it overestimates the measured porosity values (Fig. 5f, main text). This likely arises as the model assumes that the network is composed of perfectly rigid 1D fibres. By adding a prefactor of 0.94 to Eqn. 1 we achieve excellent agreement with our AgNW porosity data (Fig. 5f, main text).

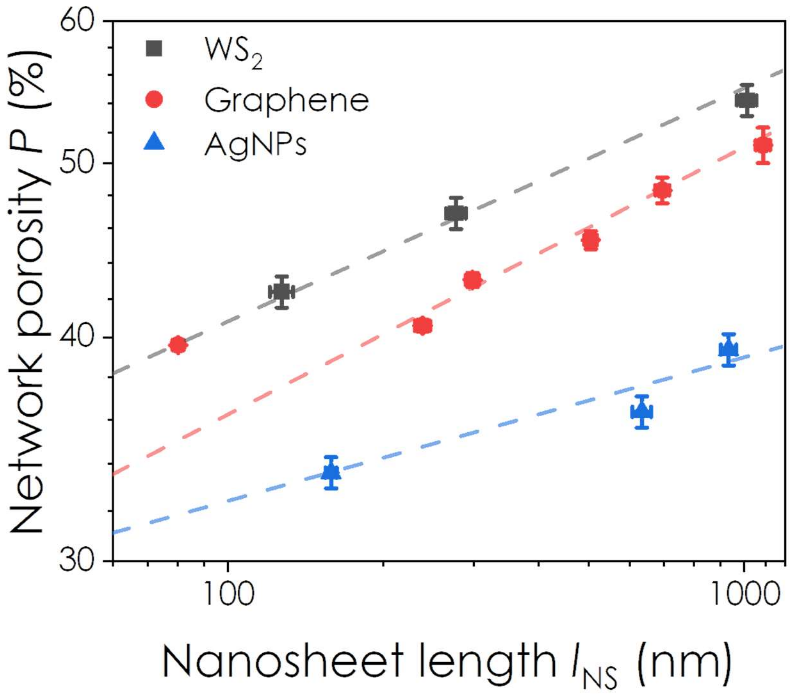

Supplementary figure 20: Comparison of network porosity, *P*, scaling with nanosheet length, l_{NS}, for the printed graphene, WS_2 and AgNS networks. The data are presented as means ± SE in the mean for nanosheet length (n = 139 - 375) and ± the root sum of squares (RSS) of segmentation error and SE in the mean for *P* (n = 175 – 701).

Supplementary note 7. Network tortuosity factor measurements

The diffusional tortuosity factor, κ, of the segmented volumes in this work was calculated using the TauFactor application in MatLab[17]. Tortuosity, τ, is used to describe the resistance of a structure to an applied flux due to a convolution of its geometry in a given direction. In this work we use the tortuosity factor, $\kappa = \tau^2$, as it has been shown to account for both the additional distance travelled and change in velocity of a species when travelling through a heterogenous structure. TauFactor calculates the reduction in diffusive transport through a network due to the presence of other phases according to

$$D_{\mathrm{eff}} = D_0 \frac{V_f}{\kappa} \quad (2)$$

where D_{eff} is the effective diffusivity of the phase in the network, D_0 is the intrinsic diffusivity of the phase, and V_{f} is the volume fraction of that phase in the network. The program calculates κ in the in-plane (x, z) and out-of-plane (y) directions for each phase in the network (i.e. pore volume and nanosheets). Crucially, it has been reported that diffusional, electrical, and thermal tortuosities are interchangeable due to the comparable underlying transport physics of each[18]. TauFactor was used to calculate the porosity, specific surface area and tortuosity factor of each 3D image. The program also generates 3D flux maps for each network so that areas with high flux can easily be visualised (Fig. 2c, main text).

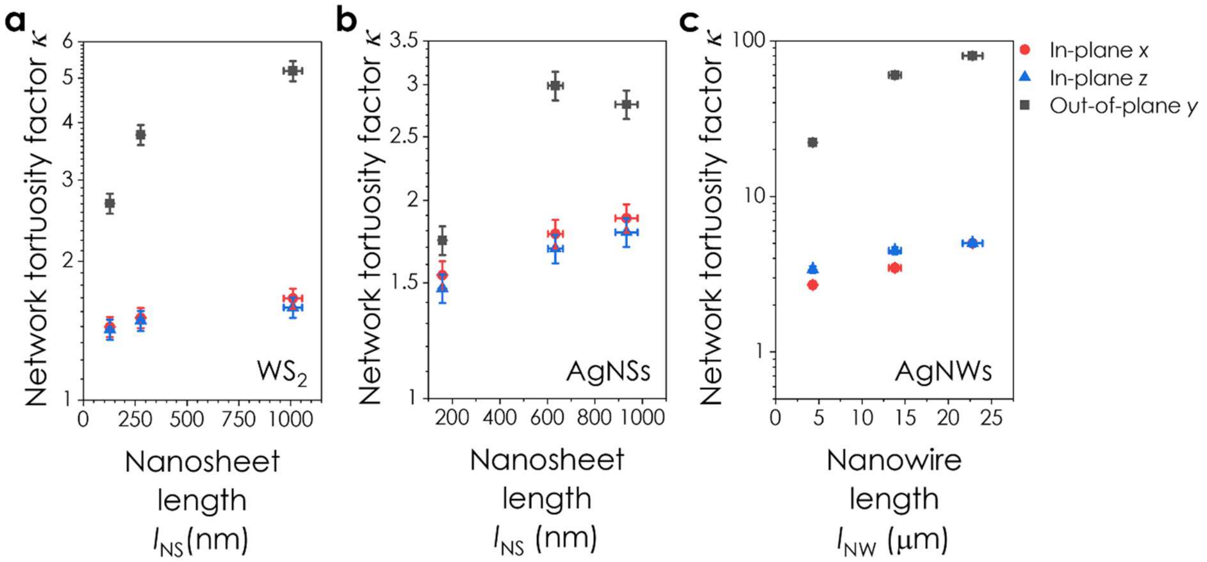

Supplementary figure 21: Nanosheet/nanowire tortuosity factor, κ, for the WS_2. AgNS and AgNW networks. Plots of the network κ in the in-plane (x,z) and out-of-plane (y) directions as a function of nanosheet length, l_{NS}, for the printed **(a)** WS_2 and **(b)** AgNS networks, and l_{NW} for the **(c)** AgNWs networks. The data are presented as means ± SE in the mean for nanosheet length (n = 139 - 375) and ± the segmentation error for κ.

The most widely utilised relationship between tortuosity factor, κ, and volume fraction, V_f, is the Bruggeman relationship[19], which has been expanded to a more generalised form[20] given by

$$\kappa = \alpha V_f^{1-\beta} \qquad (3)$$

where β is related to the shape of the objects that make up the network and α is a scaling prefactor[21]. The Bruggeman relationship is often quoted with $\alpha = 1$ and $\beta = 1.5$. However, this relation only applies for isotropic networks of spherical particles and experimentally measured exponents are often >1.5[22], with values of $\beta = 2$ - 5 predicted for high-aspect ratio particles[23]. Values of β and α were calculated for the nanosheet and pore volumes for each material in the in-plane (x,z) and out-of-plane (y) directions using Eqn. 3 (Suppl. Table 1). While there are currently no reported β-values for nanosheet networks, the calculated value of $\beta = 5.6$ for the pore volume in printed graphene networks (out-of-plane, y-direction) is larger than reported values for graphite-based electrodes measured using X-ray tomography data (≈ 500 nm voxel size)[22]. However, it has been shown that higher resolution volumes lead to increased tortuosity factor values[24].

			**Bruggeman exponent, β**	**Prefactor, α**
**Graphene**	Pores	In-plane	3.3 ± 0.8	0.4 ± 0.2
		Out-of-plane	5.6 ± 0.4	0.4 ± .02
	Nanosheets	In-plane	1.9 ± 0.5	0.9 ±1.4
		Out-of-plane	2.5 ± 0.3	1.2 ± 2.6
**WS_2**	Pores	In-plane	2.8 ± 1.1	0.6 ± 0.5
		Out-of-plane	3.3 ± 0.6	1.3 ± 0.7
	Nanosheets	In-plane	1.55 ± 0.04	1.1 ± 0.4
		Out-of-plane	3.8 ± 0.5	0.8 ± 0.5
**AgNSs**	Pores	In-plane	5.6 ± 3.2	0.007 ± .005
		Out-of-plane	6.5 ± 0.8	0.020 ± .004
	Nanosheets	In-plane	3.2 ±1.3	1.3 ± 1.2
		Out-of-plane	6.1 ± 4.1	4 ± 5
**AgNWs**	Pores	In-plane	2.1 ± 0.8	1 ± 2
		Out-of-plane	2.8 ± 0.3	1 ± 0.6
	Nanowires	In-plane	1.7 ± 0.5	1 ± 1
		Out-of-plane	2.9 ± 0.2	1 ± 3

Supplementary table 1: Experimentally measured values for the Bruggeman exponent, β, and scaling prefactor, α, in the in-plane (x, z) and out-of-plane (y) directions for the reconstructed pore and nanosheet volumes for each material. Error in α and β is the uncertainty value from fitting the Bruggeman equation to the κ vs. V_f data for each material.

While limited comparisons can be made using the nanosheet network data at present, the Bruggeman equation can be altered to consider randomly-packed rigid cylindrical particles, predicting values of $\beta = 2$ and $\alpha = 1$[19]. This agrees well with the in-plane AgNW pore data in Suppl. Table 1, where the fitted exponents were found to be $\beta \approx 2$ and $\alpha \approx 1$. The larger exponent of $\beta = 2.8$ in the out-of-plane (y) direction likely arises due to the anisotropy of the AgNW network, where nanowires are primarily aligned in the plane of the film (Fig. 5c, main text), as well as deviations from the ideal rigid cylinder assumption of the model.

Supplementary note 8. Pore size and shape

As discussed in Supplementary note 6, the pore volume in each reconstructed network was found to be highly connected, with > 99% of the total pore volume contained in a continuous macropore spanning the network. This limits 3D statistical analysis on pore shape/volume in these networks as most of the information is contained in a single data point (pore). By separating the pore volume into individual 2D cross-sections we performed statistical analysis on both the cross-sectional area and shape of the discrete pore chambers that make up the pore volume (Suppl. Fig. 22).

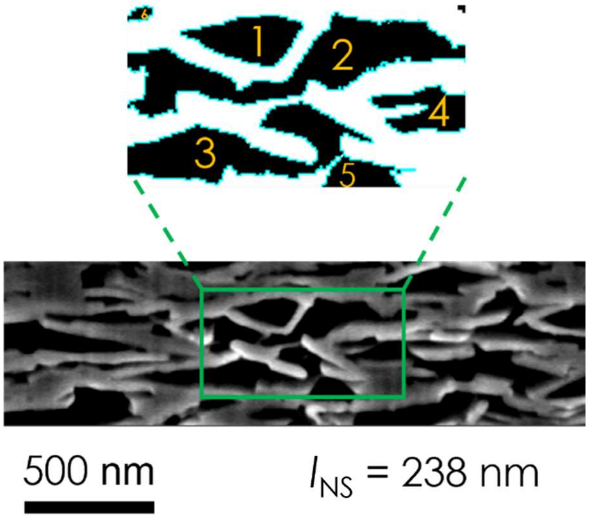

Supplementary figure 22: Pore characterisation in a printed graphene network. Pore shape and size analysis performed using the Analyse Particles plugin in FIJI[2]. Isolated pores (black) in a network cross-section are identified and labelled numerically. Nanosheets are white. The cross-sectional area, *A*, perimeter, *p*, and circularity, *C*, of each pore can then be determined.

Each network cross-section was converted to a binarised image containing only pore information. The Analyse Particles plugin in FIJI[2] was then used to identify and label each individual pore in the network slice, as shown in Suppl. Fig. 22. This program then measures the cross-sectional area, A, perimeter, p, and circularity, C, of each pore in the image. Circularity is a measure of how close in shape a given 2D object is to a circle, where C=1 describes a circle, and values <1 denote more irregular and elongated shapes. C is defined as

$$C = \frac{4\pi A}{p^2} \tag{4}$$

We measured A and C for pores in each cross-section for the printed LPE graphene networks. 3D frequency histograms (50 × 50 bins) were generated to determine the number of pores in each bin, N_{bin}, for a given pore cross-sectional area, A_{bin}, and circularity, C_{bin}. By finding the total pore area contained in each bin ($N_{\text{bin}} \times A_{\text{bin}}$) and dividing by the total area of all pores in the network we found the fraction of overall pore area, f_a, contained in each bin through

$$f_a = \frac{N_{\text{bin}} A_{\text{bin}}}{\sum_{All\ \text{bins}} N_{\text{bin}} A_{\text{bin}}} \tag{5}$$

Area-weighted heat-maps of pore cross-sectional area, A, and circularity, C, are shown in Suppl. Fig. 23 for each of the printed LPE graphene networks. The data clearly shows pore

chambers with large cross-sectional area and low circularity (high aspect ratio) to dominate the pore volume, where 90% of the total pore area is contained within the approximately linear purple bands in Suppl. Fig. 23.

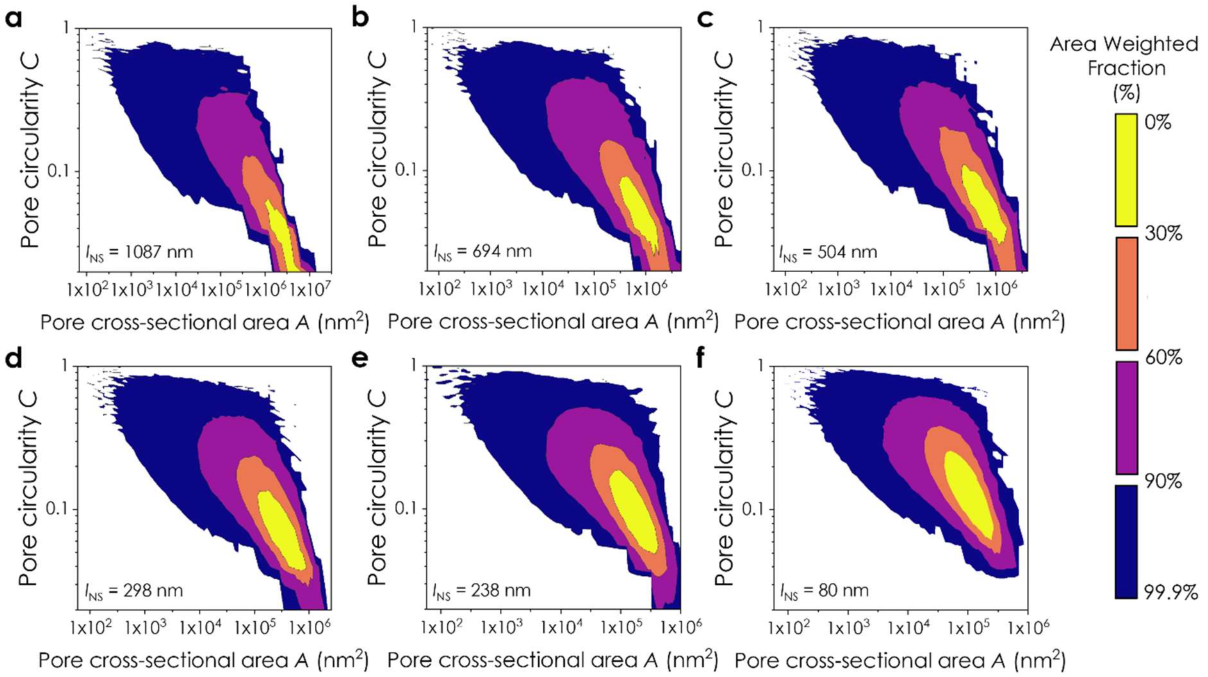

Supplementary figure 23: **Pore size and shape distributions for printed graphene networks**. **(a-f)** Contour plots showing the area-weighted pore fraction as a function of pore cross-sectional area, A, and circularity, C, for the size-selected LPE graphene networks. The distributions **(a-f)** are sorted by decreasing nanosheet length, l_{NS}.

As >90% of the total pore area was contained in the linear (purple) regions of each distribution, we isolated this data for analysis (Suppl. Fig. 24a). Here, the distributions are seen to shift to higher cross-sectional areas and lower circularities as the nanosheet size in each network was increased. The mean circularity for each nanosheet size is seen to decrease from $\approx 0.32 - 0.15$ as l_{NS} increases from 80 – 1087 nm (Suppl. Fig. 24c). The mean cross-sectional area, A, and pore size, $\xi = \sqrt{A}$, show the opposite trend where the pore dimensions are seen to increase with l_{NS} (Suppl. Fig. 24a,d). This data suggests that graphene networks composed of smaller nanosheets have smaller, more circular pores than networks composed of larger nanosheets.

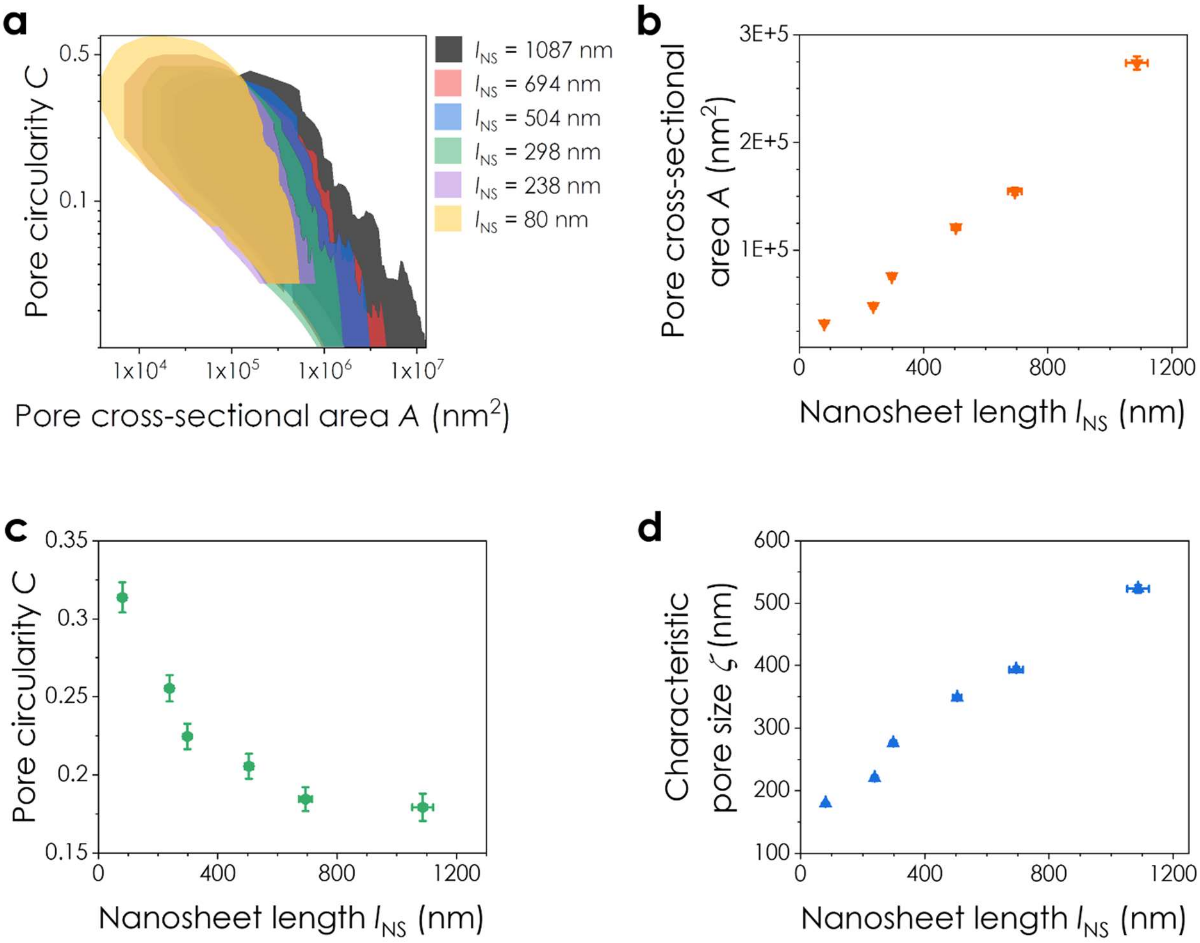

Supplementary figure 24: **Pore size and shape analysis. (a)** Distributions of pore cross-sectional area, *A*, and circularity, *C*, for the size selected LPE graphene networks where only the linear green regions of Suppl. Fig. 23 are considered. **(b)** Mean pore cross-sectional area plotted as a function of l_{NS}. **(c)** Average *C* plotted as a function of nanosheet length, l_{NS}. **(d)** Scaling of the characteristic pore size, $\xi = \sqrt{A}$, with l_{NS}. The data are presented as means ± SE in the mean for l_{NS} (n = 190 - 270) and ± the RSS of segmentation error and SE in the mean for *A*, *C* and ζ (n = 145 – 438).

The histograms used to calculate the mean pore cross-sectional area and circularity are shown in Suppl. Fig. 25 and Suppl. Fig. 26, respectively.

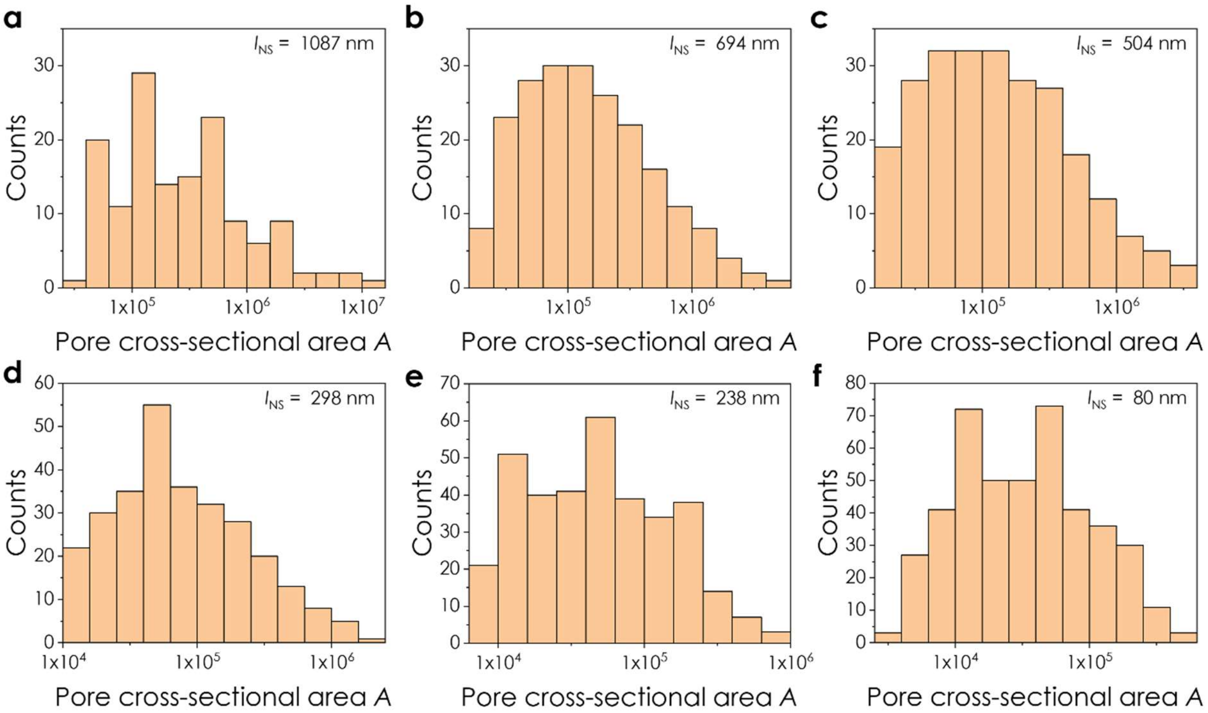

Supplementary figure 25: **Pore cross-sectional area distributions for size-selected graphene networks**. **(a-f)** Distributions of the measured pore cross-sectional area, *A*, for each printed LPE graphene network. The distributions **(a-f)** are sorted by decreasing nanosheet length, l_{NS}.

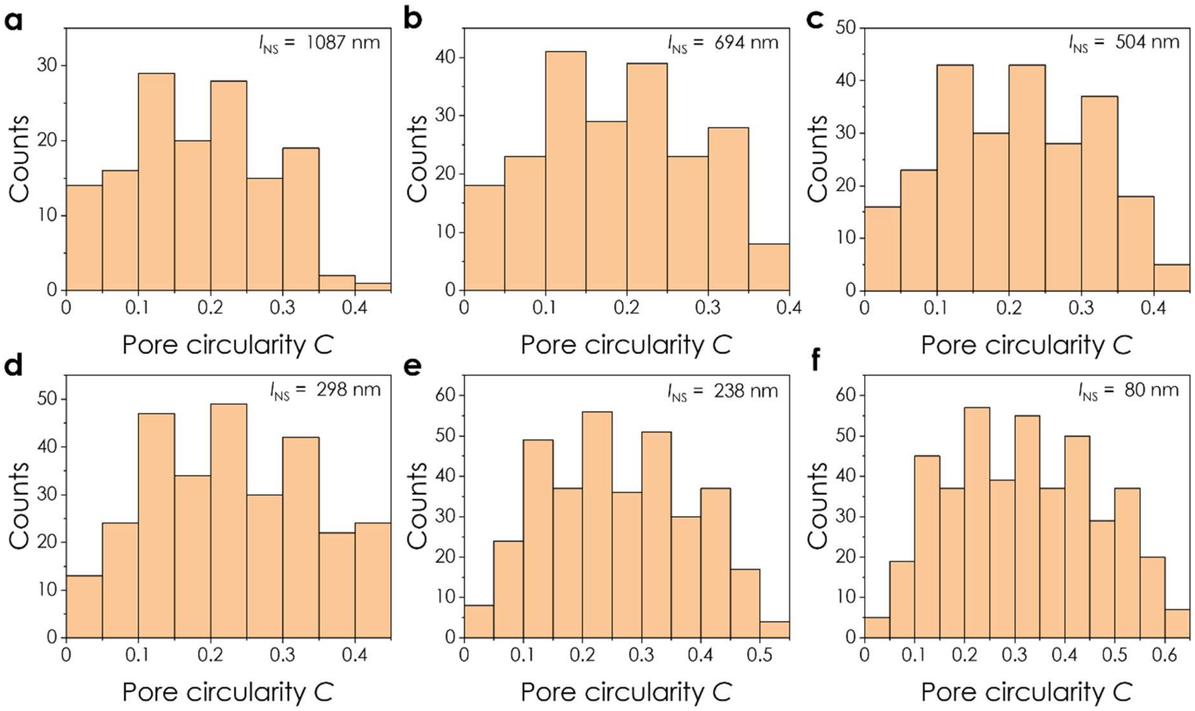

Supplementary figure 26: **Pore circularity distributions for size-selected graphene networks. (a-f)** Distributions of the measured pore circularity, C, for each printed LPE graphene network. The distributions **(a-f)** are sorted by decreasing nanosheet length, l_{NS}.

Supplementary note 9. Nanosheet aggregation within printed networks

The length and thickness of the aggregated nanosheets in the printed networks (given by l_{Net} and t_{Net} in the main text) was approximated using the Ridge Detection plugin in FIJI[25]. This approach identifies discrete contours/lines in a 2D image by using first and second directional derivatives to determine what pixels lie on the same line. The implementation of this plugin is shown on a cross-section of a printed LPE graphene network (l_{NS} = 238 nm) in Suppl. Fig. 27. Individual nanosheets in the network are approximated by red contours, the junctions between them are denoted by hollow circles and the estimated nanosheet thickness by green lines. This method was applied to multiple cross-sections for each network to extract distributions for the approximate length (l_{Net}) and thickness (t_{Net}) of the aggregated nanosheets within.

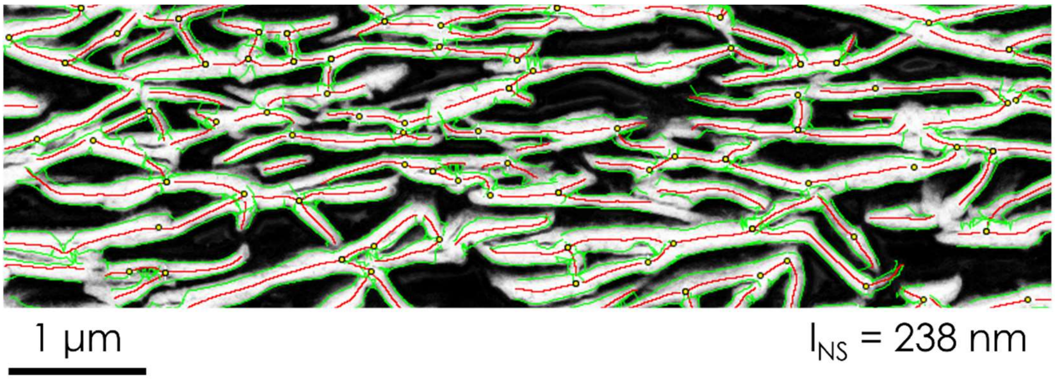

Supplementary figure 27: Nanosheet thickness and length in printed networks. Ridge Detection plugin[2,25] applied to a cross-section from a printed LPE graphene network to determine the aggregated nanosheet dimensions post-deposition. Nanosheets and pores are coloured white and black respectively. The estimated nanosheet length (l_{Net}) is denoted by red contours, inter-sheet junctions by hollow circles, and the nanosheet thickness (t_{Net}) by green lines.

The Ridge Detection algorithm requires a predefined thickness range for the nanosheets it is attempting to identify. The thickness of >100 nanosheets was manually measured from 10 cross-sections spanning the image stack for each network. To ensure each network was sampled across comparable length scales - the largest LPE graphene nanosheets were 1087 nm (or ≈72 network slices) long, while the smallest were 80 nm (or ≈ 5 network slices) long - each network was sampled at slice intervals that corresponded to 0.25 × l_{NS} for that network. The extracted thickness histograms for each of the size-selected LPE graphene networks are shown in Suppl. Fig. 28. The mean nanosheet thickness, t_{Net}, for the printed graphene networks in the main text (Figs. 2e and 4b) was calculated from the normal distributions in Suppl. Fig. 28. Both t_{Net} and the AFM measured nanosheet thickness in the ink, t_{NS}, are given for each network.

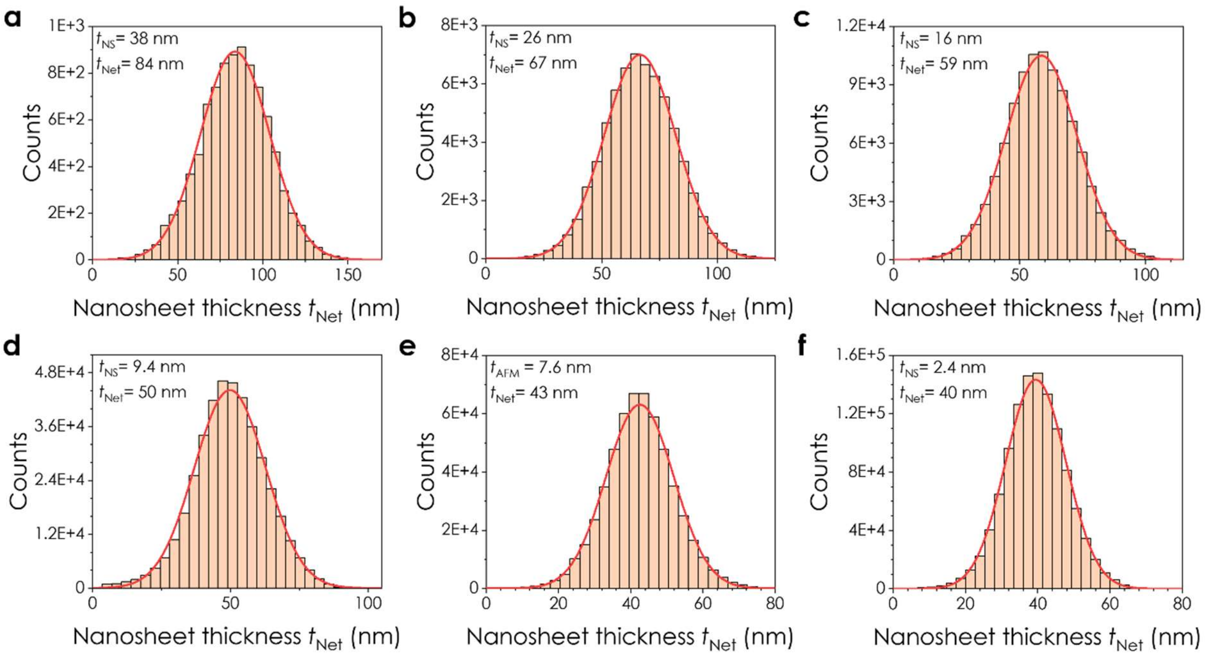

Supplementary figure 28: Distributions of nanosheet thickness, t_{Net}, within each printed LPE graphene network post-deposition. The thickness of the aggregated nanosheets was measured from network cross-sections using the Ridge Detection plugin in FIJI[10,34]. The distributions **(a-f)** are ordered in decreasing nanosheet thickness. The red lines are fits to a normal distribution. The mean nanosheet thickness in each ink, t_{NS}, and the corresponding nanosheet thickness in each network, t_{Net}, are indicated in each plot.

While measuring thickness is procedurally straightforward, additional care must be taken when using Ridge Detection to approximate the nanosheet length, l_{Net}, from 2D cross-sections. When measuring nanosheet dimensions using AFM, the longest dimension of the nanosheet is denoted as the length, l_{NS}, while the perpendicular direction is denoted as width. The Ridge Detection plugin measures the apparent length of each nanosheet as it appears in a 2D cross-section, where it is non-trivial to determine if the nanosheet length visible represents this maximised value. To prevent an underestimation of l_{Net}, the nanosheet length data was filtered to remove any nanosheet lengths that were $< 0.75 \times l_{NS}$, the nanosheet length measured by AFM. The resulting l_{Net} distributions are shown in Suppl. Fig. 29.

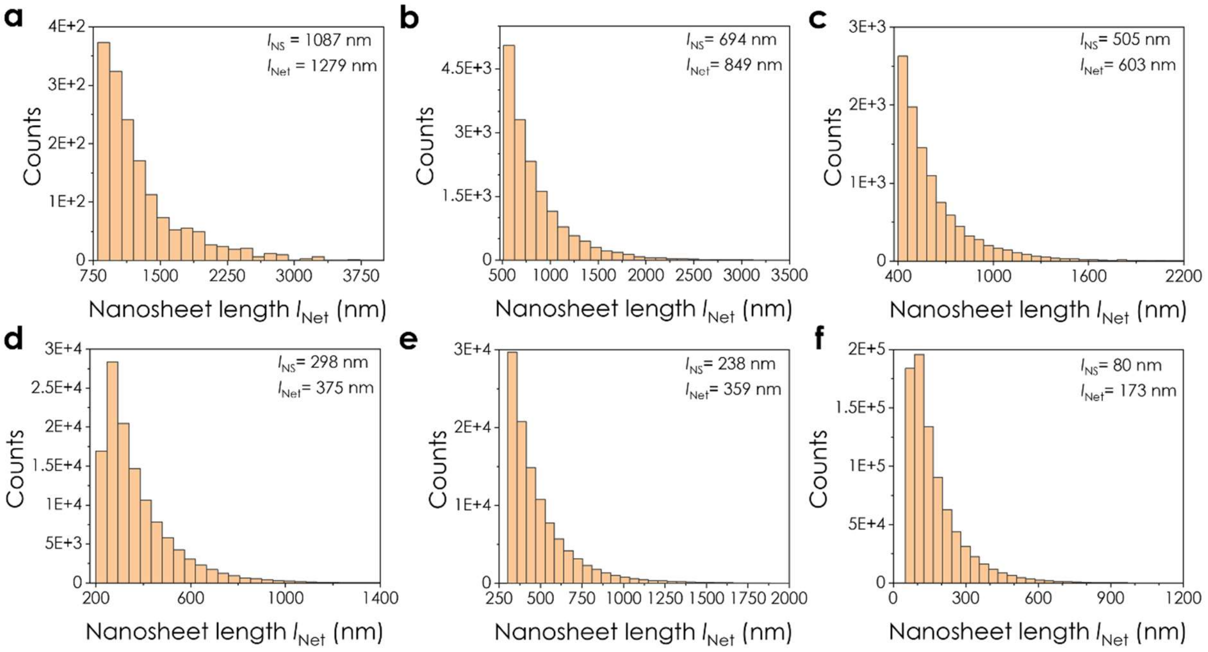

Supplementary figure 29: Distributions of nanosheet length, l_{Net}, within each printed LPE graphene network post-deposition. The length of the aggregated nanosheets was measured from network cross-sections using the Ridge Detection plugin in FIJI[10,34]. The distributions **(a-f)** are ordered in decreasing nanosheet length. The mean nanosheet length in each ink, l_{NS}, and the corresponding nanosheet length in each network, l_{Net}, are indicated in each plot.

From the data in Suppl. Figs. 28 & 29 the degree of nanosheet aggregation that occurred during film formation was approximated. The nanosheet thickness and length in the network (t_{Net}, l_{Net}) were found to scale with the AFM measured nanosheet thickness and length in the ink (t_{NS}, l_{NS}) for the LPE graphene networks (Suppl. Fig. 30a). To quantify the degree of nanosheet aggregation/restacking during film deposition, we define the thickness and length aggregation factors as χ_t and χ_l where

$$\chi_t = \frac{t_{Net}}{t_{NS}} \quad \text{and} \quad \chi_l = \frac{l_{Net}}{l_{NS}} \qquad \text{(6) and (7)}$$

For each of the size-selected printed LPE graphene networks both χ_t and χ_l were found to be >1 (Suppl. Fig. 30b). Interestingly, the data in Suppl. Fig. 30b implies that nanosheet aggregation primarily occurs by vertical restacking as χ_l was found to be only marginally greater than 1 for each network, while χ_t was found to be much larger. This basal-plane stacking is demonstrated in the AgNS networks (Fig. 5b, main text), where junctions between the individual AgNSs are more clearly visible. Furthermore, smaller nanosheets demonstrate increased aggregation when compared to networks with larger l_{NS} values in Suppl. Fig. 30b. This is likely driven by increased mobility within the ink and a lower energy barrier to aggregation for smaller/thinner nanosheets.

This size-dependent vertical restacking of nanosheets in the networks leads to considerable changes in the nanosheet aspect ratio, $k_{Net} = l_{Net}/t_{Net}$, post deposition (Suppl. Fig. 30c). Here, k_{Net} is no longer constant as it was in the ink ($k_{NS} = 29$, Fig. 3a, main text), but now exhibits a well-defined decrease from ≈16 to ≈4 as the ink nanosheet length l_{NS} was reduced from 1087 to 80 nm.

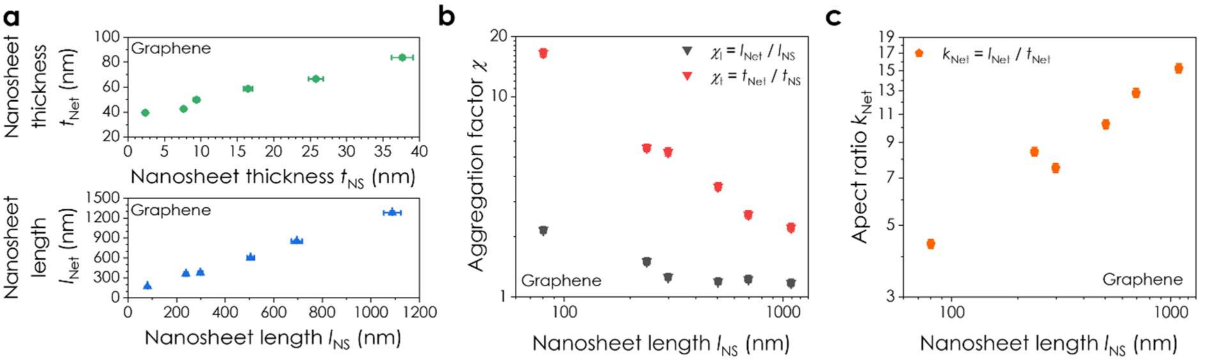

Supplementary figure 30: Nanosheet aggregation in printed graphene networks. (a) Plot of the nanosheet thickness and length in the network (t_{Net}, l_{Net}) versus the AFM measured nanosheet thickness and length in the ink (t_{NS}, l_{NS}) for the LPE graphene networks. The data are presented as means ± SE in the mean for (l_{NS}, t_{NS}) (n = 190 - 270) and ± the RSS of segmentation error and SE in the mean for (l_{Net}, t_{Net}) (n > 9000). **(b)** Plot of the aggregation factor, χ, for both nanosheet length, χ_l, and thickness, χ_t, in the printed graphene networks as a function of l_{NS}. **(c)** Measured nanosheet aspect ratio, k_{Net}, in the printed graphene networks plotted against l_{NS}. The data are presented as means ± SE in the mean for l_{NS} (n = 190 - 270) and ± the propagated error for χ according to Eqns. 6 and 7.

Similar measurements of l_{Net} and t_{Net} were performed for the printed LPE WS_2 networks, with the distributions given in Suppl. Fig. 31.

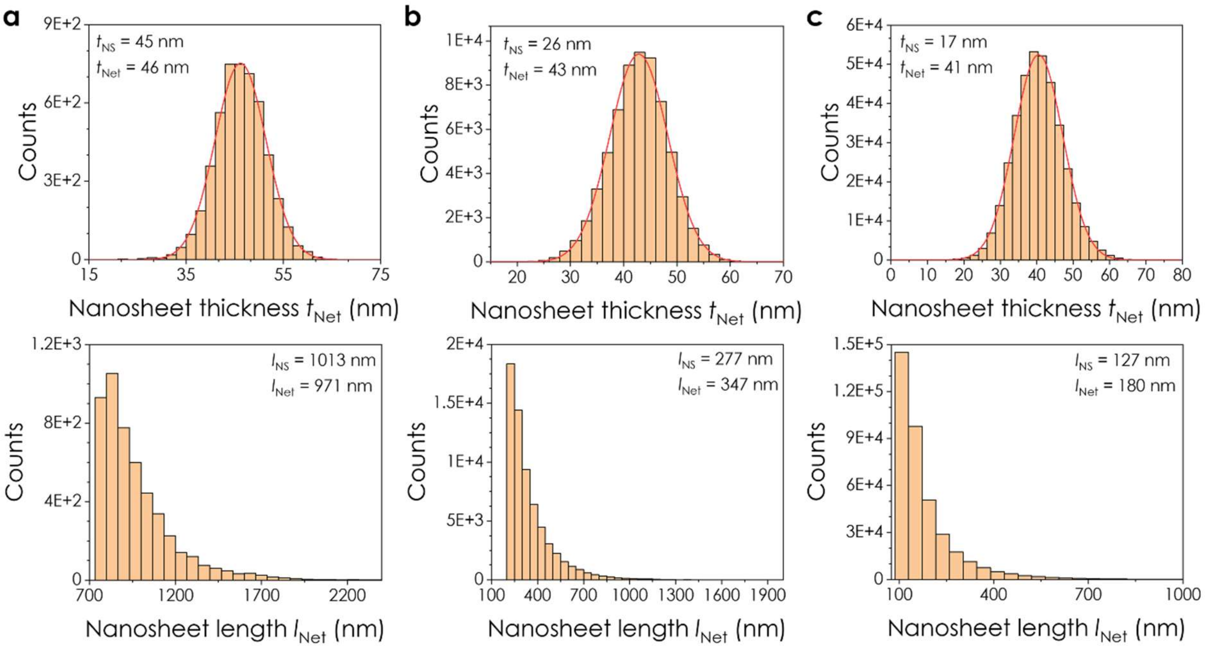

Supplementary figure 31: **Distributions of nanosheet thickness, t_{Net}, (top row) and length, l_{Net}, (bottom row) for each printed LPE WS_2 network post-deposition.** The thickness and length of the aggregated nanosheets was measured from network cross-sections using the Ridge Detection plugin in FIJI[10,34]. The distribution pairs **(a-c)** are ordered in decreasing nanosheet size. The mean nanosheet dimensions in each ink (l_{NS}, t_{NS}) and the corresponding nanosheet dimension in each network (l_{Net}, t_{Net}) are indicated in each plot. The red lines in the t_{Net} distributions are fits to a normal distribution.

The WS_2 data behaves in a similar manner to the printed graphene networks, where the nanosheets are seen to aggregate primarily through vertical restacking (Suppl. Fig. 32). This is reflected by the length and thickness aggregation factors, χ_l and χ_t, both being greater than 1, but with $\chi_t > \chi_l$ for each size-selected network. As with the graphene data in Suppl. Fig. 30b, networks of smaller nanosheets demonstrate increased levels of aggregation when compared to networks of larger LPE WS_2 nanosheets.

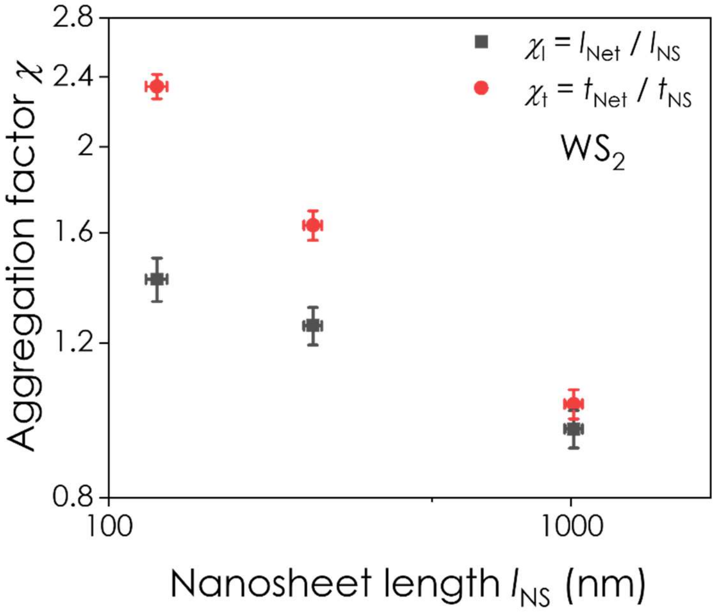

Supplementary figure 32: Nanosheet aggregation in printed WS_2 networks. Plot of the nanosheet aggregation factor, χ, for both nanosheet length, χ_l, and thickness, χ_t, in the printed WS_2 networks as a function of l_{NS}. The data are presented as means ± SE in the mean for l_{NS} (n = 259 - 375) and ± the propagated error for χ according to Eqns. 6 and 7.

Supplementary note 10. Nanosheet orientation

The Hermans orientation factor, *S*, was used to describe nanosheet orientation within the printed networks in this work[26]. This term describes the degree of alignment within a network through the angle its constituent nanosheets' normal vectors make with the substrate normal vector, φ. The Hermans orientation factor is given by

$$S = \frac{3\langle \cos^2(\phi)\rangle - 1}{2} \quad (8)$$

S=1 describes nanosheets that are perfectly aligned in the plane of the film. A value of *S*=-0.5 means the nanosheets are perfectly aligned perpendicular to the substrate and *S*=0 describes randomly orientated objects. We calculated *S* from 3D images by finding the normal vector describing each nanosheet's orientation within the network. Discrete 2D objects were first identified using a 3D Distance Transform Watershed operation from the MorphoLibJ library in FIJI[2,27]. This involved artificially introducing junctions between connected nanosheets using spatial intensity relationships between pixels[28,29]. A chessboard distance transform was used as it equally weights all directions and has shown improved performance for elongated particles when compared to other distance transforms[28]. The degree of segmentation is controlled by the dynamic setting in the program, where higher dynamic values lead to less identified objects and lower dynamic settings lead to more introduced junctions and objects (Suppl. Fig. 33). Dynamic values of 1, 2 and 3 were used to segment each network volume. The voxel connectivity was set to 26 neighbours, meaning that adjacent voxels could be connected by edges and vertices.

As shown in Suppl. Fig. 33, a dynamic setting of 1 oversegments the network, splitting nanosheets into smaller portions. This can cause *S* to be understated for the network. Alternatively, a dynamic setting of 3 is seen to undersegment the network, merging multiple nanosheets together, which can lead to an overstated *S* value (Suppl. Fig. 33). A dynamic setting of 2 led to the most accurate segmentation for each network. The values of *S* generated from networks segmented using a dynamic of 2 were taken as the true values, while the dynamic 1 and 3 values were used to estimate the lower and upper bounds of uncertainty in *S*, respectively (Fig. 4e, main text).

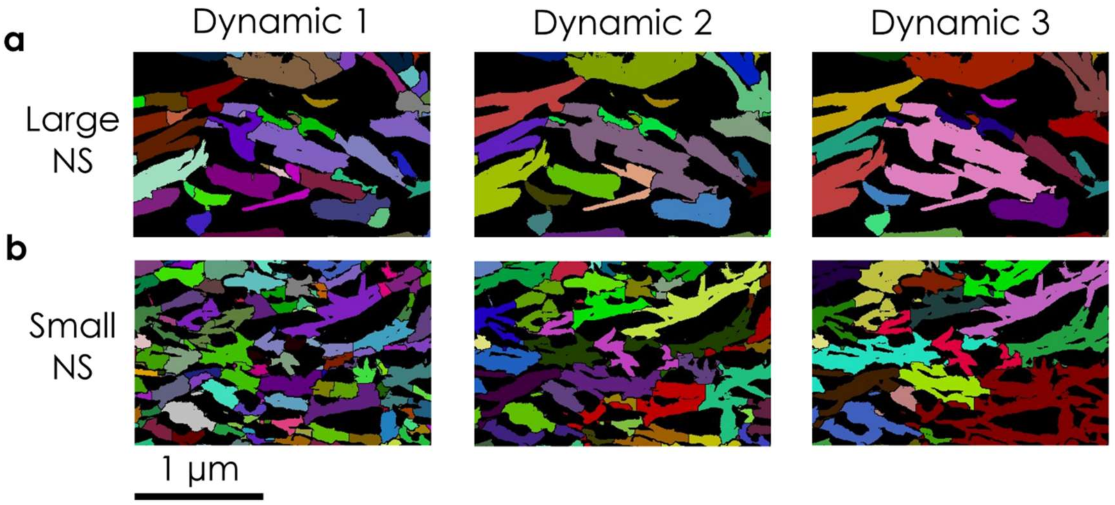

Supplementary figure 33: Nanosheet identification in 3D volumes. Segmented images generated using the 3D distance transform watershed algorithm[27] for the **(a)** largest (l_{NS} = 1087 nm) and **(b)** smallest (l_{NS} = 80 nm) graphene nanosheet networks. Each column was segmented using a different dynamic setting. Each colour represents a discrete nanosheet identified using the algorithm.

While the 3D Distance Transform Watershed performs well in grouping nanosheets with the same orientation using a dynamic setting of 2 (Suppl. Fig. 33), the segmented images were not accurate enough to allow the number of inter-nanosheet junctions or nanosheet length/thickness to be determined. However, reasonable estimates of S were calculated. A 3D image of a representative volume that has been segmented into its colour-coded nanosheet components is shown in Suppl. Fig. 34a. To calculate the orientation of the identified 2D objects, each was converted to an equivalent ellipsoid using the Equivalent Ellipsoid plugin in MorphoLibJ[27] (Suppl. Fig. 34b), which then calculates its size and orientation. The azimuth of the projection of each ellipsoid's major axis onto the XY-plane, φ, corresponds to the angle that the nanosheet normal vector makes with the Y-axis (out-of-plane direction) (Suppl. Fig. 34c).

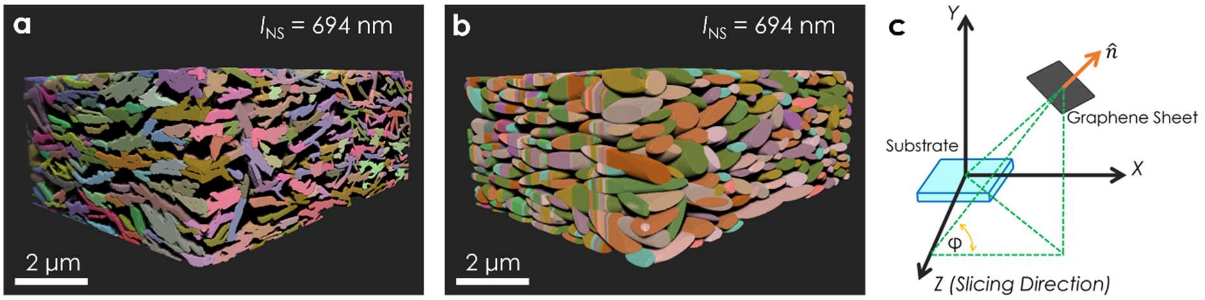

Supplementary figure 34: Nanosheet orientation analysis. (a) 3D image of a portion of a printed LPE graphene network (l_{NS} = 694 nm) segmented into its discrete colour-coded nanosheet components. **(b)** 3D volume showing the equivalent ellipsoids for each nanosheet component from **(a)**. **(c)** Schematic showing how the nanosheet normal vector makes an angle φ with the Y-axis (out-of-plane) direction.

We measured φ for each identified ellipsoid/nanosheet in the size-selected printed LPE graphene networks. Distributions of the measured angle (φ) between the nanosheet normal vectors and the out-of-plane (y) direction in each network are shown in Suppl. Fig. 35.

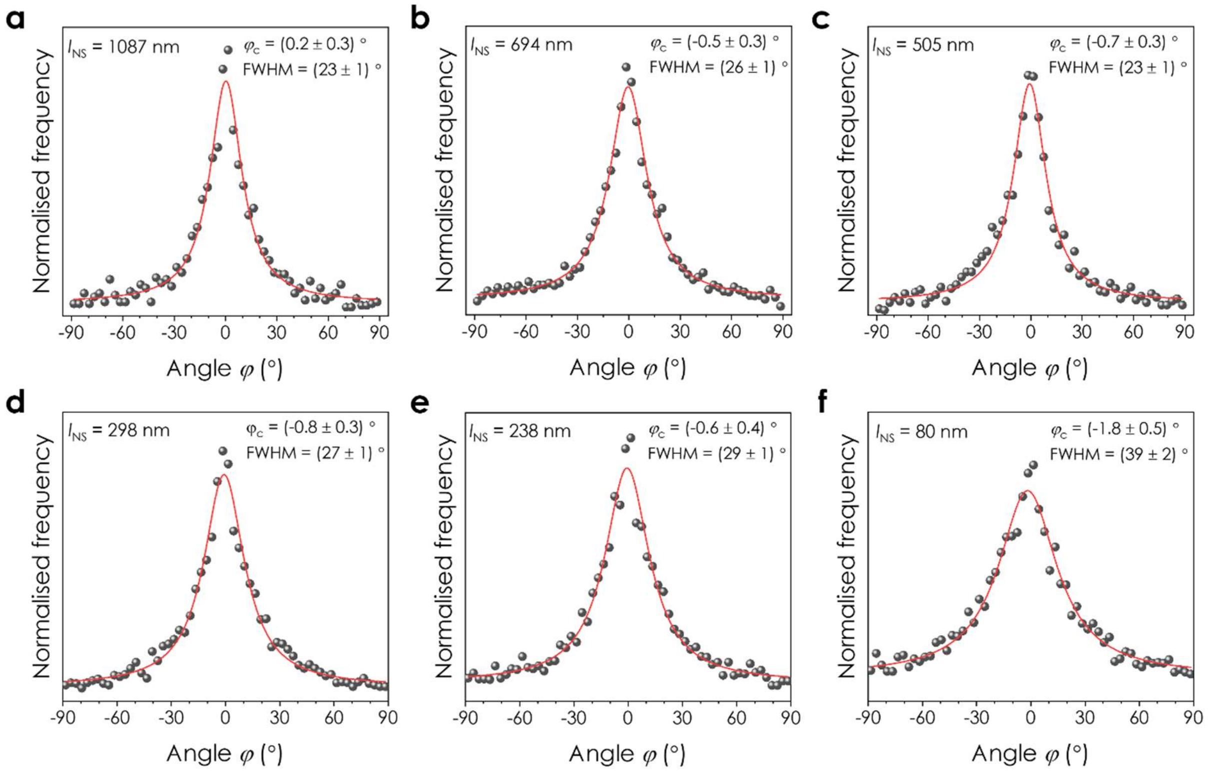

Supplementary figure 35: Nanosheet alignment and Hermans orientation factor. Distributions of the measured angle (φ) between the nanosheet normal vectors and the out-of-plane (y) direction in each of the printed LPE graphene networks. The solid lines are fits to a Cauchy-Lorentz distribution. The peak centre, φ_c, and FWHM are indicated on each plot. The distributions **(a-f)** are sorted by decreasing nanosheet length, l_{NS}.

The distributions in Suppl. Fig. 35 were fit with a Cauchy-Lorentz function to calculate the angle corresponding to the peak of the curve, φ_c, and the full width half maximum (FWHM)[30]. All distributions were centred on $\varphi_c \approx 0^\circ$ suggesting that the nanosheets are primarily aligned in the plane of the film for each network. The FWHM values give an estimate of the degree of alignment about φ_c in each film. The FWHM of $(29 \pm 1)^\circ$ for the l_{NS} = 238 nm network (Suppl. Fig. 35e) is comparable to a value of 21° measured for an inkjet-printed graphene film (l_{NS} = 208 nm) using AFM[31], and 17° measured using individual cross-sections of an uncompressed vacuum filtered graphene network (l_{NS} = 260 nm)[32]. The Hermans orientation factor was calculated using Eqn. 8 and the extracted φ-values for each network.

To corroborate the calculated φ distributions from the 3D images, the nanosheet orientation within individual 2D cross-sections was calculated using the OrientationJ plugin in FIJI[33,34]. This method has been used to calculate the alignment of 3D printed graphene nanosheets from X-ray CT data (using 2D slices)[35,36] and from fracture cross-sections of vacuum filtered graphene films[32]. We find reasonable agreement in the angular distributions generated using both techniques for small and large nanosheet networks (Suppl. Fig. 36). This suggests that the 3D distance transform watershed approach adopted here is a viable means to estimate the nanosheet orientation in 3D.

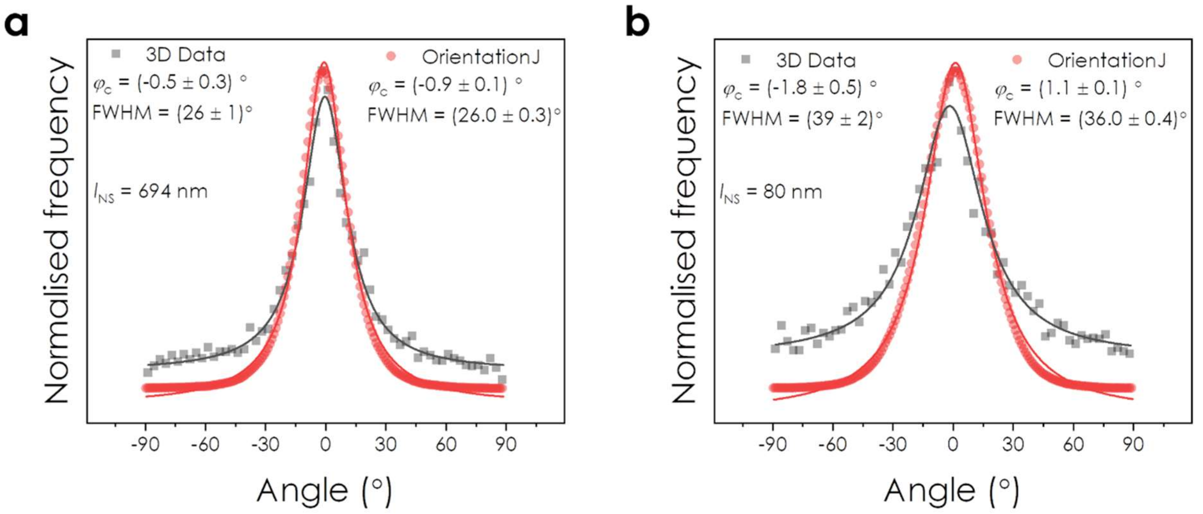

Supplementary figure 36: Comparison of nanosheet orientation analysis in 2D and 3D. Distributions of the measured angle between the nanosheet normal vectors and the out-of-plane (y) direction (black line), and the angle each nanosheet makes with the substrate in a 2D cross-section (red line), in printed LPE graphene networks where **(a)** l_{NS} = 694 nm and **(b)** l_{NS} = 80 nm. The solid lines are fits to a Cauchy-Lorentz distribution. The peak centre, φ_c, and FWHM are indicated on each plot. The uncertainty in φ_c and the FWHM is calculated from fitting the data with a Cauchy-Lorentz function.

Supplementary note 11. Electrical characterisation of printed graphene networks

The electrical properties of the size-selected printed graphene networks were measured from IV-curves for different electrode separations using two-terminal electrical measurements (Suppl. Fig. 37a). The specific contact resistivity, ρ_C, and in-plane network resistivity, ρ_{IP}, were calculated from transmission line plots for each network (Suppl. Fig. 37b). The network resistivity is plotted as a function of nanosheet length, l_{NS}, in Suppl. Fig. 37c, where ρ_{IP} is seen to increase with increasing l_{NS}. The decrease in ρ_C with increasing nanosheet volume fraction in Suppl. Fig. 37d suggests that densely-packed networks of smaller nanosheets are forming more intimate electrical contacts with the evaporated gold bottom electrodes.

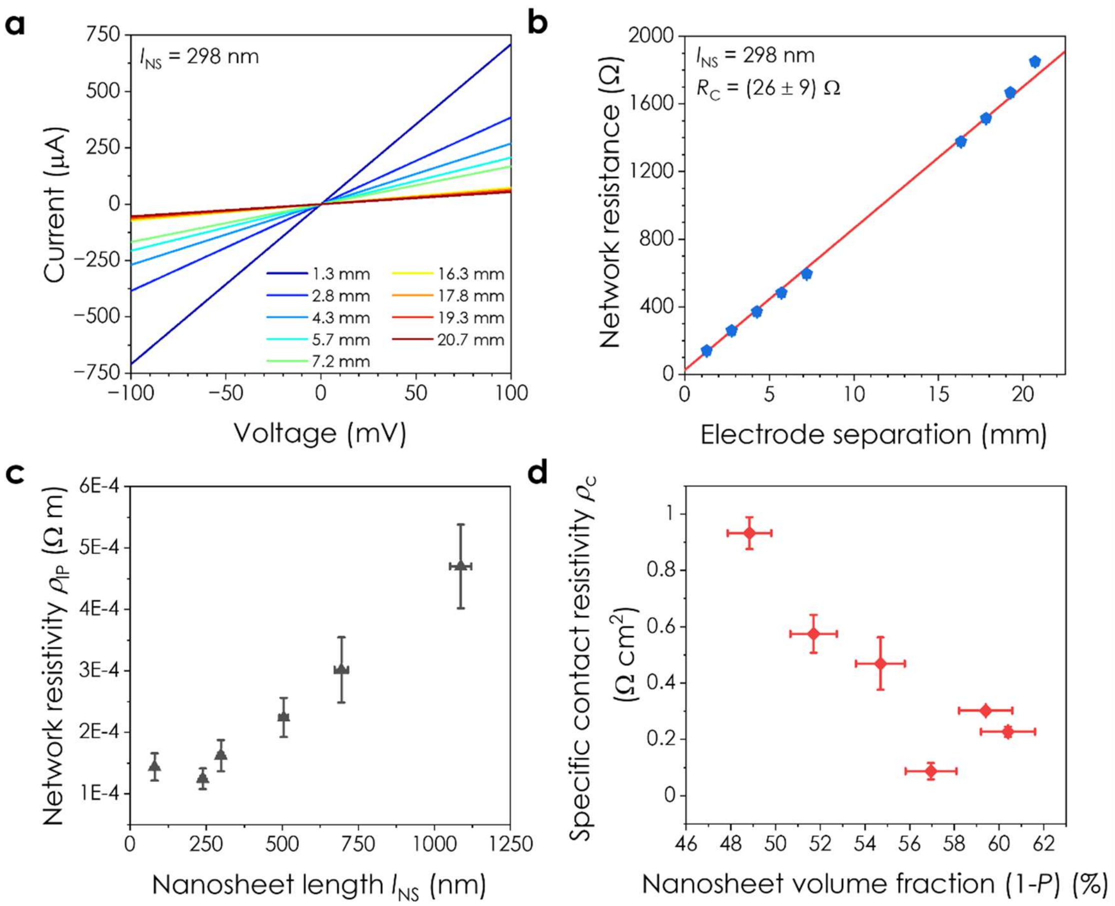

Supplementary figure 37: Electrical characterisation of printed LPE graphene networks. (a) IV-curves for a printed LPE graphene network (l_{NS} = 298 nm) for a range of electrode separations. **(b)** Plot of network resistance as a function of electrode separation for the l_{NS} = 298 nm graphene network. The solid red line is a linear fit to the data used to extract the network in-plane resistivity, ρ_{IP}, and contact resistance, R_C. The error in R_C is calculated from the linear fit. **(c)** Network resistivity, ρ_{IP}, as a function of nanosheet length, l_{NS}. The data are presented as means ± SE in the mean for l_{NS} (n = 190 - 270) and ± SE in the mean for ρ_{IP} (n = 9). (**d)** Plot of the specific contact resistivity, ρ_C, versus nanosheet volume fraction in each network. The data are presented as means ± the RSS of segmentation error and SE in the mean for nanosheet volume fraction (n = 605 - 860) and ± the fitting error for ρ_c in the transmission line plots.

Kelly et al.[37], have shown that the in-plane conductivity, σ_{IP}, of a purely junction-limited network is given by

$$\sigma_{IP} \approx (1-P)/t_{NS}R_J \qquad (9)$$

where P is the network porosity, t_{NS} is the nanosheet thickness and R_J is the junction resistance. We can include the effect of the nanosheet resistance by noting that every time an electron passes through an inter-nanosheet junction, it must subsequently pass through a nanosheet to reach the next junction. This means that R_J should be replaced by (R_J + R_{NS}) in the above equation[37]. In addition, the effect of tortuosity can be incorporated by dividing the (1-P) term (which represents the nanosheet volume fraction) by the in-plane tortuosity factor associated with the nanosheet network, κ_{IP}. This is in-line with the Bruggeman-type approach used in porous electrochemical systems where the in-pore electrolyte conductivity is related to the bulk electrolyte conductivity by $\sigma_{Pore} = \sigma_{Bulk}\varepsilon/\kappa$ where ε is the pore volume fraction and κ is the pore tortuosity factor[18].

These modifications yield the following equation for network conductivity

$$\sigma_{IP} \approx \frac{(1-P)/\kappa_{IP}}{t_{NS}(R_J+R_{NS})} \qquad (10)$$

Modelling the nanosheets as cuboids with equal length and width allows us to write

$$R_{NS} = 1/\sigma_{NS}t_{NS} \qquad (11)$$

This yields

$$\sigma_{IP} \approx \frac{\sigma_{NS}(1-P)/\kappa_{IP}}{(1+R_J\sigma_{NS}t_{NS})} \qquad (12)$$

For LPE nanosheets, the nanosheet thickness scales roughly with nanosheet length, l_{NS}, such that the aspect ratio, k, is constant ($k_{NS} = l_{NS}/t_{NS}$)[38].

$$\sigma_{IP} \approx \frac{\sigma_{NS}(1-P)/\kappa_{IP}}{(1+R_J\sigma_{NS}l_{NS}/k_{NS})} \qquad (13)$$

Converting to resistivity

$$\rho_{IP} \approx \frac{\rho_{NS}+R_J l_{NS}/k_{NS}}{(1-P)/\kappa_{IP}} \qquad (14)$$

Once, l_{NS}, ρ_{IP}, P and κ_{IP} are known, as they are here, this equation can be linearised as follows

$$\frac{\rho_{IP}(1-P)}{\kappa_{IP}} = \rho_{NS} + \frac{R_J l_{NS}}{k_{NS}} \qquad (15)$$

This means plotting $\rho_{IP}(1-P)/\kappa_{IP}$ versus l_{NS} should yield a straight line. This is plotted in Fig. 4f in the main text with very good linearity observed and yields a nanosheet resistivity of ρ_{NS} = 31 ± 2 μΩ m and junction resistance of R_J = 3.0 ± 0.4 kΩ.

To determine why the EE graphene nanosheet network is considerably more conductive than the LPE graphene network a purely junction limited model was used[37].

$$\sigma_{IP} \approx \frac{(1-P)}{\kappa_{IP}t_{NS}R_J} \qquad (16)$$

This model assumes that the resistance of the nanosheets is negligible relative to the resistance of the junctions. Taking the thickness of the LPE and EE nanosheets measured by AFM (Suppl. Fig. 9a-b) and the values reported in the main text (Fig. 6a) this equation yields a junction resistance value of 2.5 ± 0.3 kΩ for the LPE network and 3.7 ± 0.6 kΩ for the EE network. This suggests that the EE network is more conductive than the LPE network because the nanosheets are thinner and not because EE sheets form junctions with lower resistances.

Supplementary note 12. Analysis of network surfaces and interfaces

The network surface roughness (Fig. 6a, main text), as well as the interfacial roughness within printed graphene/silver nanoparticle heterostacks (Fig. 6b, main text), was calculated from the segmented network volumes. For the LPE vs. EE comparison (Fig. 6a, main text), the segmented nanosheet volumes were resliced in the *xz*-plane (top down view of the network), where each slice has a thickness of 5 nm. The Z-stack Depth Colorcode plugin in FIJI[2] was then used to encode depth information into the slice-by-slice data by assigning a different greyscale pixel intensity (from 255 to 0) to each sequential slice from the top surface of the network down. Each intensity value corresponds to a vertical height of 5 nm, meaning it is possible to reconstruct the height profile of a network surface where the peak-to-trough height is < 1275 nm. For networks where the roughness is > 1275 nm, the networks can be resliced with larger thicknesses (i.e. 10 nm slice thickness facilitates a peak-to-trough measurement of 2550 nm). Within the Z-stack Depth Colorcode plugin, an inverted grays lookup table (LUT) was used to colour code the slices and the output stack format was set as colour (RGB). The result is a stack of slices in the *xz*-plane where the highest point of the network surface has an intensity value of 255, which decreases by a value of 1 with each subsequent slice in the direction of the substrate. This stack can then be reconstructed in 3D to create a topographic map of the network surface.

The colour-coded RGB images (which had the same intensity in each channel) were converted to 8-bit greyscale and exported to the Volume Viewer plugin in FIJI[2] where an image of the depth-coded network surface was captured. Depth-coded surface images of the printed EE and LPE networks are shown in Suppl. Figs. 38a & 38b respectively. The highest surface features are coded white (pixel intensity ≈255), while the lowest parts of the surface are coded black (pixel intensity → 0). These images were used to generate a matrix of pixel intensity/surface height vs. position for each network in MatLab and their surface topography was reconstructed in 3D (Fig. 6a, main text). Measurements of surface height were performed using line-scans of the depth-coded images in Suppl. Fig. 38a-b with the Plot Profile function in FIJI. Representative surface profiles for both films are shown in Suppl. Fig. 38c. The root mean square roughness, R_{RMS}, of each network surface was calculated from the average of 12 surface height line-scans (Suppl. Table 2 & Fig. 6a, main text).

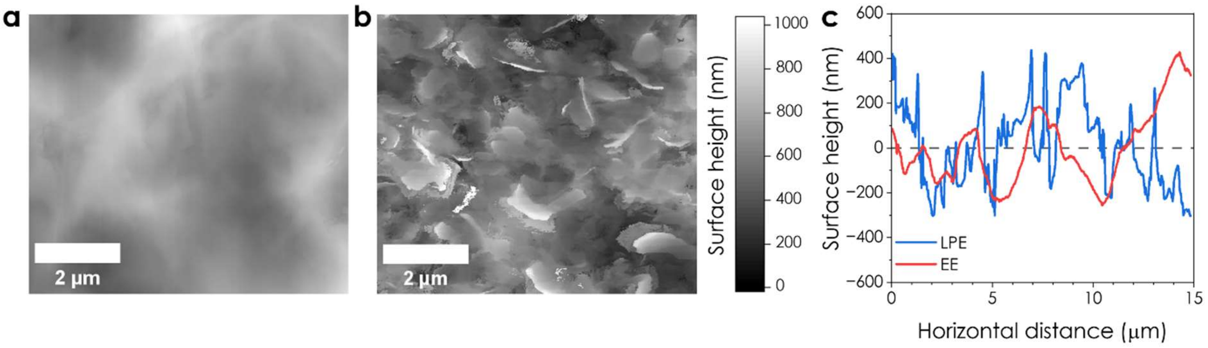

Supplementary figure 38: Surface roughness measurements for LPE and EE graphene networks. (a-b) Depth-coded greyscale surface images of portions of the **(a)** EE and **(b)** LPE films. White sections represent peaks and black sections represent valleys. **(c)** Representative surface profile line-scans of the LPE and EE films showing the difference in surface roughness. Both profiles are centred on the average surface height set at 0.

LPE surface roughness, R_{RMS} (nm)	EE surface roughness, R_{RMS} (nm)
171	165
216	158
119	75
133	147
179	119
210	119
138	141
188	70
179	127
218	106
223	114
214	101

Supplementary table 2: Measured root mean square roughness, R_{RMS}, for 12 different line scans of the printed LPE and EE graphene films.

Fig. 6b in the main text shows two graphene nanosheet networks (l_{NS}= 630 nm and l_{NS}= 215 nm) with silver nanoparticles (AgNP, diameter ≈50 nm) aerosol jet printed on the top surface. To characterise the AgNP/graphene network interface, depth-coded 3D topography maps of AgNP penetration into the nanosheet network were created (Suppl. Fig. 39a) as described above. These demonstrate the increased degree of AgNP infiltration and interfacial roughness in the network of larger (l_{NS} = 630 nm) nanosheets.

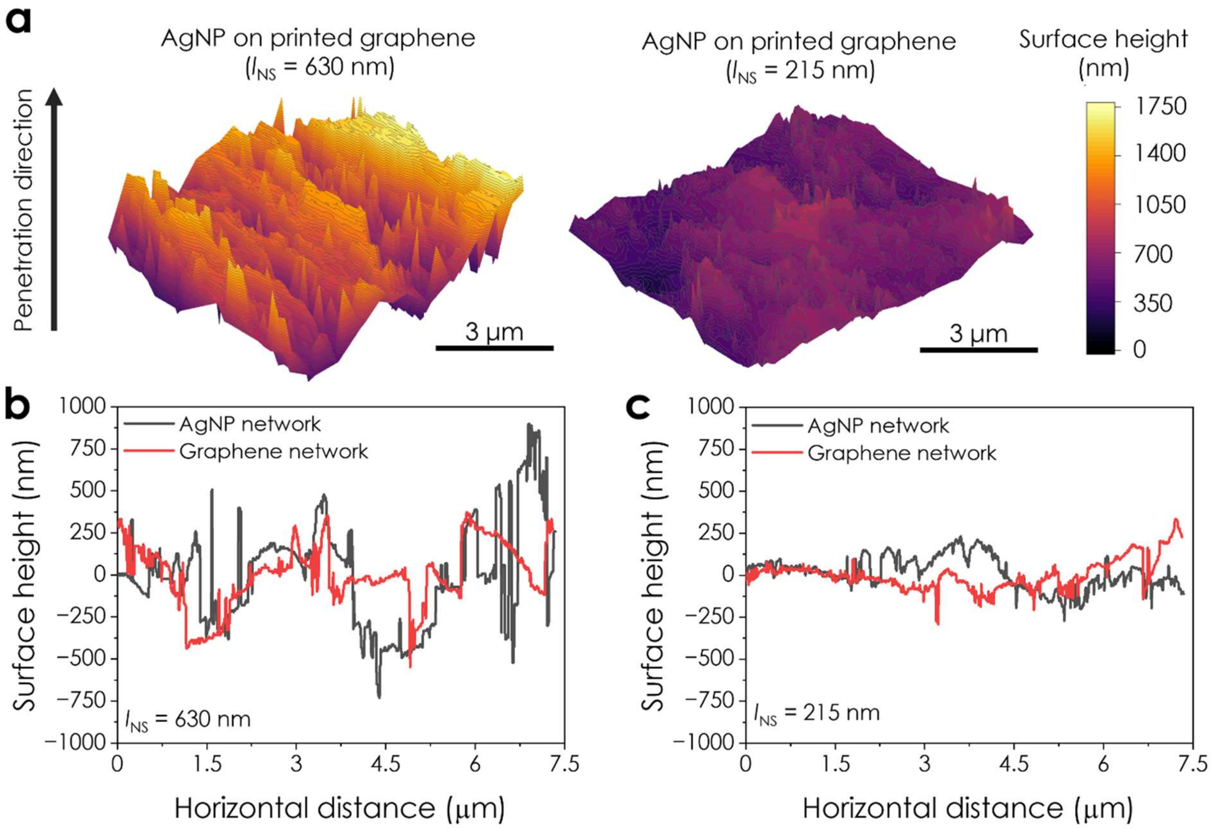

Supplementary figure 39: Interfacial analysis on printed graphene/silver nanoparticle vertical stacks. (a) 3D surface topography maps of the AgNP networks at their interface with the printed graphene networks for nanosheet lengths of l_{NS}= 630 nm and l_{NS}= 215 nm. The direction of AgNP penetration into the graphene networks is indicated and the colour scale is common to both images. **(b-c)** Representative surface profile line-scans of the AgNP and graphene networks at their interface, showing the difference in surface roughness for the **(b)** l_{NS}= 630 nm and **(c)** l_{NS}= 215 nm networks. Both profiles are centred on the average surface height set at 0.

We calculated the R_{RMS} for both the graphene and AgNP networks at their interface (Suppl. Table 3) for the l_{NS}= 630 nm and l_{NS}= 215 nm samples. The l_{NS} = 630 nm graphene network had a mean surface roughness of R_{RMS}= 158 ± 48 nm, while the AgNPs exhibited a R_{RMS} of 313 ± 55 nm. In comparison, the l_{NS}= 215 nm graphene network R_{RMS} of 105 ± 18 nm was in perfect agreement with the AgNP R_{RMS} of 102 ± 18 nm. The increased AgNP roughness for the network of larger nanosheets ($R_{AgNP}/R_{Graphene}$ ≈ 2) reflects a significant level of AgNP penetration into the graphene network. Alternatively, the agreement in surface R_{RMS} at the AgNP/graphene interface for the l_{NS}= 215 nm network suggests minimal penetration and a higher quality interface.

l_{NS} = 630 nm network		l_{NS} = 215 nm network	
**Graphene surface R_{RMS} (nm)**	**AgNP surface R_{RMS} (nm)**	**Graphene surface R_{RMS} (nm)**	**AgNP surface R_{RMS} (nm)**
177	380	99	124
166	269	121	109
103	227	112	107
71	295	97	99
75	212	103	112
178	356	103	88
175	359	121	83
159	321	105	83
197	347	146	137
187	369	97	115
221	306	89	80
189	310	73	87

Supplementary table 3: Network roughness at the graphene/AgNP interface. Measured root mean square roughness, R_{RMS}, for 12 different line scans of the printed graphene and AgNP networks at their interface for networks of large (l_{NS}= 630 nm) and small (l_{NS}= 215 nm) graphene nanosheets.

The degree of AgNP infiltration into printed graphene networks with l_{NS}= 630 nm and l_{NS}= 215 nm is shown in the main text (Fig. 6b). AgNP/graphene 3D volumes were resliced in the *xz*-plane as described above, and the volume fraction of both graphene nanosheets and AgNPs was found for each slice (Fig. 6b, main text). Using the surface topography maps in Suppl. Fig. 39a, the lowest point of the AgNP surface was determined. The Find Connected Regions plugin (described in Supplementary note 6) was utilised to find the maximum depth that an electrically percolating AgNP path penetrated into the graphene network. This distance was found to be ≈ 725 nm into the network of larger graphene nanosheets (l_{NS} = 630 nm), with no penetration into the network of smaller nanosheets (l_{NS} = 215 nm) observed.

Supplementary note 13. Depth Aware video frame INterpolation (DAIN)

One of the video frame interpolation algorithms that can be used for the resolution enhancement of FIB-SEM-NT-produced 3D volumes is Depth-Aware video frame INterpolation (DAIN)[39]. It is made of two main components: a depth estimation network, which investigates quantities such as the distance between items in the frame, and a flow estimation network, used to determine the motion of pixels between consecutive frames. DAIN's developers provide a ready-to-use Google Collaboratory notebook (URL) that can be easily accessed and run, with the possibility to use free GPUs provided by Google to accelerate the generation process. It should be noted that the model is already trained on a large dataset, namely the Vimeo90K dataset[40], and there is no need for fine-tuning. The notebook is configured to accept a video as input and to convert it into a sequence of frames. For this application, the notebook can be easily altered to accept frames (images in a stack) as input directly. After this small adjustment, the user only needs to specify the data path of the input folder, containing the experimentally captured frames, the data path of the output folder, where the new dataset will be saved, and finally the number of new frames that the model would need to generate between each pair of consecutive frames. For example, to homogenise the anisotropic 5 × 5× 15 nm voxels used in this work, DAIN could be used to generate 2 intermediate frames between each slice in the milling (z) direction to produce voxels with 5 × 5× 5 nm dimensions. The outcome is a folder containing a sequence of ordered frames, which form a volume of higher resolution compared to the original experimentally captured volume.

Supplementary references

1 Arganda-Carreras, I. *et al.* Trainable Weka Segmentation: a machine learning tool for microscopy pixel classification. *Bioinformatics* **33**, 2424-2426, doi:10.1093/bioinformatics/btx180 (2017).

2 Schindelin, J. *et al.* Fiji: an open-source platform for biological-image analysis. *Nature Methods* **9**, 676-682, doi:10.1038/nmeth.2019 (2012).

3 Yao, N. *et al.* Sampling Depth Controlled by Accelerating Voltage in a Low Voltage SEM. *Microscopy and Microanalysis* **3**, 1241-1242, doi:10.1017/s143192760001309x (2020).

4 Goldstein, J. I. *et al. Scanning Electron Microscopy and X-Ray Microanalysis*. (2018).

5 Rodenas, T. & Prieto, G. FIB-SEM tomography in catalysis and electrochemistry. *Catalysis Today* **405-406**, 2-13, doi:10.1016/j.cattod.2022.09.013 (2022).

6 Breiman, L. Random Forests. *Machine Learning* **45**, 5-32, doi:10.1023/a:1010933404324 (2001).

7 RÖDing, M. *et al.* Three-dimensional reconstruction of porous polymer films from FIB-SEM nanotomography data using random forests. *Journal of Microscopy* **281**, 76-86, doi:10.1111/jmi.12950 (2020).

8 Ridler, T., Calvard, S. Picture Thresholding Using an Iterative Selection Method. *IEEE Transactions on Systems, Man, and Cybernetics* **8**, 630-632, doi:10.1109/tsmc.1978.4310039 (1978).

9 Krygier, M. C. *et al.* Quantifying the unknown impact of segmentation uncertainty on image-based simulations. *Nature Communications* **12**, doi:10.1038/s41467-021-25493-8 (2021).

10 Yuan, H. *et al.* 4D Imaging of ZnO-Coated Nanoporous Al2O3 Aerogels by Chemically Sensitive Ptychographic Tomography: Implications for Designer Catalysts. *ACS Applied Nano Materials* **4**, 621-632, doi:10.1021/acsanm.0c02924 (2021).

11 Ivanchenko, M. V. *et al.* Serial scanning electron microscopy of anti-PKHD1L1 immuno-gold labeled mouse hair cell stereocilia bundles. *Scientific Data* **7**, doi:10.1038/s41597-020-0509-4 (2020).

12 Moon, J., Caron, J.-B. & Gaines, R. R. Synchrotron imagery of phosphatized eggs in Waptia cf. W. fieldensis from the middle Cambrian (Miaolingian, Wuliuan) Spence Shale of Utah. *Journal of Paleontology* **96**, 152-163, doi:10.1017/jpa.2021.77 (2021).

13 Berger, C. *et al.* Cryo-electron tomography on focused ion beam lamellae transforms structural cell biology. *Nature Methods* **20**, 499-511, doi:10.1038/s41592-023-01783-5 (2023).

14 Schulenburg, H. *et al.* 3D Imaging of Catalyst Support Corrosion in Polymer Electrolyte Fuel Cells. *The Journal of Physical Chemistry C* **115**, 14236-14243, doi:10.1021/jp203016u (2011).

15 Parkhouse, J. G., & Kelly, A. The random packing of fibres in three dimensions. *Proceedings of the Royal Society of London. Series A: Mathematical and Physical Sciences* **451**, 737-746, doi:10.1098/rspa.1995.0152 (1997).

16 Kim, M. J., Seo, Y., Cruz, M. A. & Wiley, B. J. Metal Nanowire Felt as a Flow-Through Electrode for High-Productivity Electrochemistry. *ACS Nano* **13**, 6998-7009, doi:10.1021/acsnano.9b02058 (2019).

17 Cooper, S. J., Bertei, A., Shearing, P. R., Kilner, J. A. & Brandon, N. P. TauFactor: An open-source application for calculating tortuosity factors from tomographic data. *SoftwareX* **5**, 203-210, doi:10.1016/j.softx.2016.09.002 (2016).

18 Fu, J., Thomas, H. R. & Li, C. Tortuosity of porous media: Image analysis and physical simulation. *Earth-Science Reviews* **212**, doi:10.1016/j.earscirev.2020.103439 (2021).

19 Tjaden, B., Cooper, S. J., Brett, D. J. L., Kramer, D. & Shearing, P. R. On the origin and application of the Bruggeman correlation for analysing transport phenomena in electrochemical systems. *Current Opinion in Chemical Engineering* **12**, 44-51, doi:10.1016/j.coche.2016.02.006 (2016).

20 Tjaden, B., Brett, D. J. L. & Shearing, P. R. Tortuosity in electrochemical devices: a review of calculation approaches. *International Materials Reviews* **63**, 47-67, doi:10.1080/09506608.2016.1249995 (2016).

21 Thorat, I. V. *et al.* Quantifying tortuosity in porous Li-ion battery materials. *Journal of Power Sources* **188**, 592-600, doi:10.1016/j.jpowsour.2008.12.032 (2009).

22 Kehrwald, D., Shearing, P. R., Brandon, N. P., Sinha, P. K. & Harris, S. J. Local Tortuosity Inhomogeneities in a Lithium Battery Composite Electrode. *Journal of The Electrochemical Society* **158**, doi:10.1149/2.079112jes (2011).

23 Patel, K. K., Paulsen, J. M. & Desilvestro, J. Numerical simulation of porous networks in relation to battery electrodes and separators. *Journal of Power Sources* **122**, 144-152, doi:10.1016/s0378-7753(03)00399-9 (2003).

24 Landesfeind, J., Ebner, M., Eldiven, A., Wood, V. & Gasteiger, H. A. Tortuosity of Battery Electrodes: Validation of Impedance-Derived Values and Critical Comparison with 3D Tomography. *Journal of The Electrochemical Society* **165**, A469-A476, doi:10.1149/2.0231803jes (2018).

25 Steger, C. An unbiased detector of curvilinear structures. *IEEE Transactions on Pattern Analysis and Machine Intelligence* **20**, 113-125, doi:10.1109/34.659930 (1998).

26 Hermans, J. J., Hermans, P. H., Vermaas, D. & Weidinger, A. Quantitative evaluation of orientation in cellulose fibres from the X-ray fibre diagram. *Recueil des Travaux Chimiques des Pays-Bas* **65**, 427-447, doi:10.1002/recl.19460650605 (1946).

27 Legland, D., Arganda-Carreras, I. & Andrey, P. MorphoLibJ: integrated library and plugins for mathematical morphology with ImageJ. *Bioinformatics*, doi:10.1093/bioinformatics/btw413 (2016).

28 Chen, Q., Yang, X. & Petriu, E. M. in *Proceedings of the 3rd IEEE international workshop on haptic, audio and visual environments and their applications* Vol. 2 111-116 (2004).

29 Ghoshal, D. & PratimAcharjya, P. Watershed Segmentation based on Distance Transform and Edge Detection Techniques. *International Journal of Computer Applications* **52**, 6-10, doi:10.5120/8259-1792 (2012).

30 Lin, X. *et al.* Fabrication of Highly-Aligned, Conductive, and Strong Graphene Papers Using Ultralarge Graphene Oxide Sheets. *ACS Nano* **6**, 10708-10719, doi:10.1021/nn303904z (2012).

31 Piatti, E. *et al.* Charge transport mechanisms in inkjet-printed thin-film transistors based on two-dimensional materials. *Nature Electronics* **4**, 893-905, doi:10.1038/s41928-021-00684-9 (2021).

32 Barwich, S. *et al.* On the relationship between morphology and conductivity in nanosheet networks. *Carbon* **171**, 306-319, doi:10.1016/j.carbon.2020.09.015 (2021).

33 Püspöki, Z., Storath, M., Sage, D. & Unser, M. in *Focus on Bio-Image Informatics Advances in Anatomy, Embryology and Cell Biology* Ch. Chapter 3, 69-93 (2016).

34 Rezakhaniha, R. *et al.* Experimental investigation of collagen waviness and orientation in the arterial adventitia using confocal laser scanning microscopy. *Biomechanics and Modeling in Mechanobiology* **11**, 461-473, doi:10.1007/s10237-011-0325-z (2011).

35 Ding, H., Barg, S. & Derby, B. Direct 3D printing of graphene using capillary suspensions. *Nanoscale* **12**, 11440-11447, doi:10.1039/c9nr10831a (2020).

36 Tagliaferri, S. *et al.* Aqueous Inks of Pristine Graphene for 3D Printed Microsupercapacitors with High Capacitance. *ACS Nano* **15**, 15342-15353, doi:10.1021/acsnano.1c06535 (2021).

37 Kelly, A. G., O'Suilleabhain, D., Gabbett, C. & Coleman, J. N. The electrical conductivity of solution-processed nanosheet networks. *Nature Reviews Materials* **7**, 217-234, doi:10.1038/s41578-021-00386-w (2021).

38 Backes, C. *et al.* Equipartition of Energy Defines the Size–Thickness Relationship in Liquid-Exfoliated Nanosheets. *ACS Nano* **13**, 7050-7061, doi:10.1021/acsnano.9b02234 (2019).

39 Bao, W. *et al.* in *Proceedings of the IEEE/CVF Conference on Computer Vision and Pattern Recognition.* 3703-3712.

40 Xue, T., Chen, B., Wu, J., Wei, D. & Freeman, W. T. Video Enhancement with Task-Oriented Flow. *International Journal of Computer Vision* **127**, 1106-1125, doi:10.1007/s11263-018-01144-2 (2019).
