# Supplementary material for: Quantitative analysis of printed nanostructured networks using high-resolution 3D FIB-SEM nanotomography
Source: Nat Commun. 2024 Jan 4;15:278. doi: 10.1038/s41467-023-44450-1 (PMC10767099; doi:10.1038/s41467-023-44450-1)
Supplement: Supplementary file 3 — Description of Additional Supplementary Files [file 41467_2023_44450_MOESM3_ESM.pdf]

Description of Additional Supplementary Files

File Name: Supplementary Movie 1
Description: **Printed LPE Graphene Network Reconstructed using FIB-SEM-NT.**
Animation showing the internal structure of a printed LPE graphene network (l_{NS} = 695 nm). The 706 μm^3 network portion is split into its pore volume (orange) and nanosheet (white) contributions to demonstrate the morphology of each at both high and low magnification.
