# Supplementary material for: Quantitative analysis of printed nanostructured networks using high-resolution 3D FIB-SEM nanotomography
Source: Nat Commun. 2024 Jan 4;15:278. doi: 10.1038/s41467-023-44450-1 (PMC10767099; doi:10.1038/s41467-023-44450-1)
Supplement: Supplementary file 5 — Reporting Summary [file 41467_2023_44450_MOESM5_ESM.pdf]

nature portfolio

Corresponding author(s): Jonathan N. Coleman

Last updated by author(s): Nov 24, 2023

Reporting Summary

Nature Portfolio wishes to improve the reproducibility of the work that we publish. This form provides structure for consistency and transparency in reporting. For further information on Nature Portfolio policies, see our Editorial Policies and the Editorial Policy Checklist.

Statistics

For all statistical analyses, confirm that the following items are present in the figure legend, table legend, main text, or Methods section.

n/a	Confirmed	
☐	☒	The exact sample size (*n*) for each experimental group/condition, given as a discrete number and unit of measurement
☐	☒	A statement on whether measurements were taken from distinct samples or whether the same sample was measured repeatedly
☒	☐	The statistical test(s) used AND whether they are one- or two-sided<br>*Only common tests should be described solely by name; describe more complex techniques in the Methods section.*
☒	☐	A description of all covariates tested
☒	☐	A description of any assumptions or corrections, such as tests of normality and adjustment for multiple comparisons
☐	☒	A full description of the statistical parameters including central tendency (e.g. means) or other basic estimates (e.g. regression coefficient) AND variation (e.g. standard deviation) or associated estimates of uncertainty (e.g. confidence intervals)
☒	☐	For null hypothesis testing, the test statistic (e.g. *F*, *t*, *r*) with confidence intervals, effect sizes, degrees of freedom and *P* value noted<br>*Give P values as exact values whenever suitable.*
☒	☐	For Bayesian analysis, information on the choice of priors and Markov chain Monte Carlo settings
☒	☐	For hierarchical and complex designs, identification of the appropriate level for tests and full reporting of outcomes
☒	☐	Estimates of effect sizes (e.g. Cohen's *d*, Pearson's *r*), indicating how they were calculated

Our web collection on statistics for biologists contains articles on many of the points above.

Software and code

Policy information about availability of computer code

Data collection	FIB-SEM Nanotomography was performed using the ZEISS ATLAS 5 software suite (Version 5.3.3.31).
Data analysis	The generated image stacks for each network were aligned and reconstructed in 3D using Dragonfly (Version 2022.1.0.1231, Object Research Systems). Data analysis and figure plotting was performed using Origin Pro 2020 (Version 9.7.5.184). Measurements of the network tortuosity factor, specific surface area and porosity were performed using the Taufactor application (Version 1.6) in Matlab (R2021b). Image processing and analysis was performed using ImageJ (FIJI) (Version 1.54). Within ImageJ, the MorpholibJ, OrientationJ, Ridge Detection, Find Connected Regions, and Trainable WEKA Segmentation plugins were used. Computer generated intermediate slices were produced using Depth-Aware video frame INterpolation (DAIN) trained on the Vimeo90k dataset without further fine tuning.

For manuscripts utilizing custom algorithms or software that are central to the research but not yet described in published literature, software must be made available to editors and reviewers. We strongly encourage code deposition in a community repository (e.g. GitHub). See the Nature Portfolio guidelines for submitting code & software for further information.

Data

Policy information about availability of data

All manuscripts must include a data availability statement. This statement should provide the following information, where applicable:

- Accession codes, unique identifiers, or web links for publicly available datasets
- A description of any restrictions on data availability
- For clinical datasets or third party data, please ensure that the statement adheres to our policy

All data needed to evaluate the conclusions in the paper are present in the manuscript and supplementary information. The data supporting the findings of this study are also available from the corresponding author upon request. Source data are provided with this paper.

Research involving human participants, their data, or biological material

Policy information about studies with human participants or human data. See also policy information about sex, gender (identity/presentation), and sexual orientation and race, ethnicity and racism.

Reporting on sex and gender	This study does not report on human participants, their data or biological material. This also applies to reporting on sex or gender.
Reporting on race, ethnicity, or other socially relevant groupings	This study does not report on human participants, their data or biological material. This also applies to reporting on race, ethnicity, or other socially relevant groupings.
Population characteristics	This work does not involve human research participants.
Recruitment	No participants were recruited for this work.
Ethics oversight	As there was no research involving human participants, their data, or biological material, no approval of the study protocol was required.

Note that full information on the approval of the study protocol must also be provided in the manuscript.

Field-specific reporting

Please select the one below that is the best fit for your research. If you are not sure, read the appropriate sections before making your selection.

☒ Life sciences ☐ Behavioural & social sciences ☐ Ecological, evolutionary & environmental sciences

For a reference copy of the document with all sections, see nature.com/documents/nr-reporting-summary-flat.pdf

Life sciences study design

All studies must disclose on these points even when the disclosure is negative.

Sample size	This work is a systematic study to characterise the morphology of printed nanomaterial networks using FIB-SEM nanotomography. Statistical methods to predetermine same size were not required.
Data exclusions	No data were excluded from the analyses.
Replication	The experiments performed were repeated across nanomaterial networks of different types and dimensionality, where common morphological scalings with nanomaterial size were found.
Randomization	Randomization was not utilised in this work. Here, we demonstrate a technique to characterise the morphology of printed nanosheet networks and perform a systematic study to isolate the influence of nanosheet size on the morphology of these systems. All processing steps were standardised to ensure that the only variable parameter was nanosheet size.
Blinding	Blinding was not relevant to this work. Here, networks of size-selected nanosheets were specifically produced and characterised using FIB-SEM nanotomography. All processing factors were standardised to ensure that the only variable parameter was nanosheet size.

Reporting for specific materials, systems and methods

We require information from authors about some types of materials, experimental systems and methods used in many studies. Here, indicate whether each material, system or method listed is relevant to your study. If you are not sure if a list item applies to your research, read the appropriate section before selecting a response.

Materials & experimental systems

n/a	Involved in the study
☒	☐ Antibodies
☒	☐ Eukaryotic cell lines
☒	☐ Palaeontology and archaeology
☒	☐ Animals and other organisms
☒	☐ Clinical data
☒	☐ Dual use research of concern
☒	☐ Plants

Methods

n/a	Involved in the study
☒	☐ ChIP-seq
☒	☐ Flow cytometry
☒	☐ MRI-based neuroimaging

Plants

Seed stocks

Report on the source of all seed stocks or other plant material used. If applicable, state the seed stock centre and catalogue number. If plant specimens were collected from the field, describe the collection location, date and sampling procedures.

Novel plant genotypes

Describe the methods by which all novel plant genotypes were produced. This includes those generated by transgenic approaches, gene editing, chemical/radiation-based mutagenesis and hybridization. For transgenic lines, describe the transformation method, the number of independent lines analyzed and the generation upon which experiments were performed. For gene-edited lines, describe the editor used, the endogenous sequence targeted for editing, the targeting guide RNA sequence (if applicable) and how the editor was applied.

Authentication

Describe any authentication procedures for each seed stock used or novel genotype generated. Describe any experiments used to assess the effect of a mutation and, where applicable, how potential secondary effects (e.g. second site T-DNA insertions, mosiacism, off-target gene editing) were examined.
